# *Streptococcus pneumoniae* promotes migration and invasion of A549 cells *in vitro* by activating mTORC2/AKT through up-regulation of DDIT4 expression

**DOI:** 10.3389/fmicb.2022.1046226

**Published:** 2022-12-19

**Authors:** Xiaojie Song, Baohong Liu, Guanghui Zhao, Xiaoxin Pu, Baoyi Liu, Meiling Ding, Yuwen Xue

**Affiliations:** ^1^Department of Pulmonary and Critical Care Medicine, Qilu Hospital of Shandong University, Qingdao, China; ^2^Department of Hospital Infection Management, Qilu Hospital of Shandong University, Qingdao, China; ^3^Medical Laboratory Center and Oncology Laboratory, Qilu Hospital of Shandong University, Qingdao, China; ^4^Department of Infectious Diseases, Qilu Hospital of Shandong University, Qingdao, China; ^5^Department of Pulmonary and Critical Care Medicine, Qilu Hospital of Shandong University, Jinan, China

**Keywords:** lung cancer, DDIT4, mTORC1/2, AKT, migration and invasion

## Abstract

**Introduction:**

Dysbiosis of the lower airway flora is associated with lung cancer, of which the relationship between Streptococcus, especially pathogenic *Streptococcus pneumoniae* (*S. pneumoniae*), and the progression of lung cancer are unclear.

**Methods:**

Bronchoalveolar lavage fluid (BALF) samples were prospectively collected from patients with pulmonary nodules during diagnostic bronchoscopy, and finally included 70 patients diagnosed with primary lung cancer and 20 patients with benign pulmonary nodules as the disease control group. The differential flora was screened by 16S ribosomal RNA (rRNA) gene amplicon sequencing. An *in vitro* infection model of lung adenocarcinoma (LUAD) cells exposed to *S.pneumoniae* was established to observe its effects on cell migration and invasion ability. Exploring the molecular mechanisms downstream of DDIT4 through its loss- and gain-of-function experiments.

**Results:**

16S rRNA sequencing analysis showed that the abundance of Streptococcus in the lower airway flora of lung cancer patients was significantly increased. After exposure to *S. pneumoniae*, A549 and H1299 cells significantly enhanced their cell migration and invasion ability. The results of DDIT4 loss- and gain-of-function experiments in A549 cells suggest that up-regulation of DDIT4 activates the mTORC2/Akt signaling pathway, thereby enhancing the migration and invasion of A549 cells while not affecting mTORC1. Immunofluorescence (IF) and fluorescence in situ hybridization (FISH) showed that *S. pneumoniae* was enriched in LUAD tissues, and DDIT4 expression was significantly higher in cancer tissues than in non-cancerous tissues. The increased expression of DDIT4 was also related to the poor prognosis of patients with LUAD.

**Discussion:**

The data provided by this study show that *S. pneumoniae* enriched in the lower airway of patients with lung cancer can up-regulate DDIT4 expression and subsequently activate the mTORC2/AKT signal pathway, thereby increasing the migration and invasion abilities of A549 cells. Our study provides a potential new mechanism for targeted therapy of LUAD.

## Introduction

Lung cancer is a severe threat to human health. The latest cancer burden data released by the International Agency for Research on Cancer (IARC) show that lung cancer will be the second most common cancer worldwide in 2020. Still, it is the number one cancer-related cause of death among cancer patients worldwide, far surpassing other cancer types (Sung et al., 2021). Although the pathogenesis of lung cancer has been explored and the current treatment methods for lung cancer have increased significantly, the outcome of lung cancer remains unsatisfactory. Several emerging pieces of evidence suggest that dysbiosis of the lower airway flora plays a vital role in lung disease (Goto, 2022).

Healthy human lungs were once considered sterile because the vast majority of lung microorganisms could not be detected by culture (Fonkou et al., 2018). The development of high-throughput sequencing technologies subverted this perception. It is generally accepted that Firmicutes, Proteobacteria, Bacteroidetes, and Actinobacteria are the main phyla of the pulmonary microbiota (Moffatt and Cookson, 2017). Their dysbiosis is associated with a variety of lung diseases, such as cystic fibrosis (CF; Cuthbertson et al., 2020), asthma (Hufnagl et al., 2020), chronic obstructive pulmonary disease (COPD; Pragman et al., 2018), bronchiectasis (Mac Aogain et al., 2021) and lung cancer (Tsay et al., 2021).

The potential mechanisms for correlation or causation between intratumoral bacteria and tumors, including lower airway flora and lung cancer, remain unclear. Nejman et al. (2020) Performed 16S ribosomal RNA (rRNA) gene amplicon sequencing and immunohistochemistry (IHC)/Immunofluorescence (IF) analysis on more than 1,500 samples from seven tumors, including lung cancer, and found that intratumoral bacteria are present in both cancer cells and immune cells, and their composition varies according to tumor type. Jin et al. (2019) found that lung cancer progression was inhibited in germ-free mice. At the same time, pulmonary commensal flora promoted the development and progression of lung adenocarcinoma (LUAD) by activating γδ T cells in the lungs.

Notably, oral bacteria that enter the lungs through microaspiration are gaining attention. Segal et al. (2016) found that enrichment of oral bacterial taxa in the lung, such as Prevotella and Veillonella, was associated with an inflammatory phenotype, including increased levels of Th17 cells, enhanced expression of inflammatory cytokines, and a diminished TLR4 response in alveolar macrophages. Tsay et al. (2021) further showed that many oral bacteria (e.g., Streptococcus and Verotrichia) were detected in the lower airways of lung cancer patients and were associated with up-regulation of ERK and PI3K signaling pathways.

*Streptococcus pneumoniae*, an opportunistic pathogen of the genus Streptococcus, colonize the mucosal surfaces of the human upper respiratory tract (URT; Kilian and Tettelin, 2019). Up to 27–65% of children and less than 10% of adults are carriers of *S. pneumoniae*, with a complex symbiotic and pathogenic relationship with the host (Abdullahi et al., 2012; Yahiaoui et al., 2016). Lin et al. (2014) found that *S. pneumoniae* pneumonia was associated with an increased risk of lung cancer based on a population-based cohort study of over 100,000 with a hazard ratio of 3.25 [95% confidence interval (CI), 3.39–3.42]. Kooter et al. (2013) reported that *S. pneumoniae* could cause damage to alveolar epithelial cells. As one of the mechanisms, Olotu et al. (2019) showed that direct inhibition of purinergic signaling by *S. pneumoniae* through induction of phosphorylation and internalization of the purinergic receptor P2Y2 can lead to inhibition of calcium response to ATP in alveolar epithelial cells, affecting cellular integrity and function. Dong et al. (2020) provided preliminary epigenetic evidence that a variety of bacterial toxins produced by *S. pneumoniae*, including pneumolysin and pyruvate oxidase, can dephosphorylate histone H3 on serine 10 (H3S10) *via* the host cell phosphatase PP1. That increased levels of H3S10ph are associated with cancer development and transmission. However, the exact mechanism by which *S. pneumoniae* infection induces lung cancer remains elusive.

This study first identified differential bacterial taxa in bronchoalveolar lavage fluid (BALF) samples from lung cancer patients and disease controls, with Streptococcus significantly enriched in lung cancer. By sequencing the mRNA changes of A549 cells co-cultured with *S. pneumoniae*, it was found that *S. pneumoniae* infection activates mTORC2 by up-regulating the expression of DNA Damage Inducible Transcript 4 (DDIT4), which in turn activates the AKT pathway, thereby promoting the migration and invasion of A549 cells *in vitro*. Fluorescence *in situ* hybridization (FISH) and IF experiments on lung cancer tissues and adjacent tissues confirmed that *S. pneumoniae* and DDIT4 were enriched and highly expressed in lung cancer tissues, and high expression of DDIT4 was associated with poor prognosis of LUAD patients.

## Materials and methods

### Subject and public database

The 120 subjects were recruited from patients with pulmonary nodules in the Department of Respiratory Medicine, Qilu Hospital of Shandong University (Qingdao, China) from June 2019 to October 2020. The wax-embedded tissue blocks were obtained from 55 patients with primary lung cancer who underwent surgical resection from January 2016 to December 2016, and the follow-up date was up to December 2021. The approval number is KYLL-KS-qdql2018025. All the subjects signed informed consent prior to be enrolled in this study. The ethics committee of Qilu Hospital of Shandong University (Qingdao) approved this study.

### Bacterial strains and cell culture

*Streptococcus pneumoniae* (#49619) was purchased from American type culture collection (ATCC, Manassas, United States) and streaked on blood agar plates (bioMerieux, Lyon, France) at 37°C, 5% CO_2_ to obtain single colonies. Heat-killed *S.pneumoniae* was prepared by heating at 65°C for 2 h. A549 and H1299 cell lines were purchased from Zhong Qiao Xin Zhou Biotechnology Co., Ltd. (Shanghai, China) and was grown in RPMI-1640 medium (Gibco, NY, United States) supplemented with 10% fetal bovine serum (FBS, Hyclone, Logan, United States) at 37°C with 5% CO_2._ For co-culture experiments, A549 cells or H1299 cells were incubated with *S. pneumoniae* at the multiplicity of infection (MOI) of 100:1 for 4 h, and PBS served as the control group. In addition, 0.25 μg/ml oxacillin (the purity is 99.99%, Beijing, China) was added to the co-culture system to verify whether the clearance of *S.pneumoniae* affected the above biological effects.

### RNA extraction and real-time quantitative chain reaction (qPCR)

Total RNA was extracted from cells by TRIzol and then reverse transcribed into cDNA by RevertAid First Strand cDNA Synthesis Kits (Thermo Fisher Scientific, MA, United States). qPCR was performed using Blaze Taq™SYBR®Green qPCR Mix 2.0 (GeneCopoeia, Maryland, United States). The target gene expression was normalized according to the internal reference gene β-actin. The primers used in this study are as follows: DDIT4 forward, 5′-GGTTTGACCGCTCCACGAG-3′ and reverse, 5′-ATCCAGGTAAGCCGTGTCTTC-3′; β-actin forward, 5′-CTACCTCATGAAGATCCTCACCGA-3′ and reverse, 5′-TTCTCCTTAATGTCACGCACGATT-3′; Rictor forward, 5′-TTCCCTTTCTTTGCTTCT-3′ and reverse, 5′-GTGTTCTGATTCGCCTGT-3′; Raptor forward, 5′-CGTAGCCGACAAGGACAGCA-3′ and reverse, 5′-CGTCAGCAGAAGCGAGCAGT-3′.

### Western blotting

Cells were homogenized in cold RIPA buffer with phenyl methane sulfonyl fluoride (PMSF) and phosphatase inhibitor. Protein concentrations were determined by the Bicinchoninic Acid (BCA) Protein Assay Kit (Sparkjade, Jinan, China). Equal protein samples were separated by SDS-PAGE and then transferred onto PVDF membranes (Millipore, MA, United States). PVDF membranes were blocked with 5% skimmed milk powder and incubated with primary antibodies overnight at 4°C. The primary antibodies used in this study are as follows: DDIT4 (1:500; ER1706-76, HUABIO, Hangzhou, China), AKT1 (1:500; ER1609-47, HUABIO, Hangzhou, China), phosphorylated AKT1 (Ser473) (p-AKT1; 1:500; ER1607-73, HUABIO, Hangzhou, China), RP70S6KB1 (1:1,000; #2708, Cell Signaling Technology, Boston, United States), p-RP70S6KB1 (Thr389) (p-S6K; 1:1,000; #9234, Cell Signaling Technology, Boston, United States), Raptor (1:500; ER1802-57, HUABIO, Hangzhou, China), Rictor (1:500; EM1709-50, HUABIO, Hangzhou, China), and β-actin (1:1,000; R1207-1, HUABIO, Hangzhou, China). After staining with HRP-conjugated secondary antibody, protein bands were visualized using the Immobilon Western HRP Substrate Kit (Millipore, MA, United States).

### 16S rRNA sequencing

The total DNA of the microbiome was extracted by Cetyltrimethylammonium Bromide (CTAB) method, and PCR amplified the V3-V4 region with primers 341F and 805R. The PCR products were purified by AMPure XT beads (Beckman Coulter Genomics, MA, United States) and quantified by Qubit (Invitrogen, California, United States). The PCR amplification products were recovered by AMPure XT beads recovery kit (Beckman, California, United States). The purified PCR products are evaluated, and the concentration of qualified libraries should be above 2 nM. After gradient dilution, the suitable sequencing library was transformed into single strand by NaOH and then double-end sequenced at 2 × 250 bp using NovaSeq6000 sequencer (Illumina, California, United States).

### Transwell chamber experiment

A549 cells or H1299 cells were co-incubated with *S. pneumoniae* or heat-killed *S. pneumoniae* for 4 h. PBS-treated cells were used as the negative control. The cells were suspended in 200 μl serum-free medium and inoculated into the upper chamber of the Transwell chambers, and the lower chamber was filled with 500 μl medium containing 10% FBS. After 16 ~ 24 h, the cells remaining in the upper chamber were removed, and the transmigrated cells were stained with crystal violet and placed under a microscope for observation. The results of the invasion experiments were analyzed using Image J software.

### Cell scratch experiment

A549 cells or H1299 cells were inoculated in a 6-well plate, and when the cell fusion rate reached 100%, the tip of a 20 μl pipet tip was used to make a “+” scratch, and *S. pneumoniae*, heat-killed *S. pneumoniae*, and PBS were added for 4 h. After removing the co-cultures, images were taken at 0 and 24 h, respectively. Cell migration rates were analyzed using Image J software. The mean scratch width is equal to the scratch void area divided by the length, and the cell migration rate is equal to the mean scratch width at 24 h minus the mean scratch width at 0 h and then divided by the mean scratch width at 0 h, and finally multiplied by 100%.

### siRNAs and plasmid

Three siRNA are designed for DDIT4, and their sequences are as follows: siRNA1, 5′-GAGGAGUGUUGAACUUCAATT-3′; siRNA2, 5′-GAUGAACACUUGUGUGCCATT-3′; siRN.

A3, 5′-GUAGCAUGUACCUUAUUAUTT-3′. Non-targeted sequences, 5′-UUCUCCGAACGUG.

UCACGUTT-3′ were used as negative controls. All siRNAs were synthesized by GenePharma Co., Ltd. (Shanghai, China). DDIT4 overexpression plasmid was synthesized by GeneCopoeia Co., Ltd. (MD, United States). The siRNAs and plasmid were transfected into A549 cells or H1299 cells in Opti-MEM™ I reduced serum medium (Gibco, NY, United States) using lipo2000 transfection reagent (Invitrogen, California, United States).

### mRNA sequencing

A549 cells were incubated with *S. pneumoniae* or PBS for 4 h. Total RNA was extracted from A549 cells using Trizol reagent, and mRNA was sorted, and then first- and second-strand cDNA synthesis was performed on the purified RNA. After end complementation, the Illumina sequencing junction was ligated to both ends of the DNA library using T4 DNA ligase. High-fidelity polymerase was applied to amplify the original library, and after quality control, high-throughput sequencing was performed in 2 × 150 bp double-end sequencing mode using the IlluminaHiseq platform (Illumina, California, United States).

### IF and FISH

Lung cancer tissue sections were dewaxed to water on slides, boiled in repair solution, and cooled naturally; then proteinase K (20ug/ml) was added and digested for 18 min at 37°C. After washing with PBS, the pre-hybridization solution was added and incubated at 37°C for 1 h. The pre-hybridization solution was decanted, and the hybridization solution containing *the S. pneumoniae* probe was added and hybridized overnight at 42°C. After washing off the hybrid solution, serum was added to block for 30 min. *S. pneumoniae* probe, 5’-CY3-GTGATG CAAGTGCACCTT-CY3-3′, was synthesized by General Biological Co., Ltd. (Anhui, China). DDIT4 primary antibody (1:100) was added and incubated overnight at 4°C. After PBS washing, the tissue sections were incubated with FITC-labeled fluorescent secondary antibody for 50 min at room temperature, and then the nuclei were stained with DAPI. After washing, the sections were sealed with the fluorescence quenching agent and examined under a microscope.

### Immunohistochemistry

Two patients with LUAD were randomly selected by follow-up, one had survived for 5 years without recurrence, and the other died of multiple metastases in the 18th month after surgery. The wax-embedded tissue sections of the two patients were routinely dewaxed to water. After antigen repair and sealing, DDIT4 first antibody (1:50) was added and incubated at 37°C for 1 h. After washing, an HRP-linked second antibody was added and incubated at room temperature for 30 min. Finally, DAB staining was performed for 3 min.

### Statistical analysis

The 16S rRNA gene sequencing data were first subjected to data splitting, splicing, filtering, and DADA2 denoising by the applicable methods of the Illumina NovaSeq platform. *α* diversity was used to assess the within-habitat diversity, including Chao1, Observed species, Goods coverage, Shannon, Simpson. *β* diversity (between-habitat diversity) was mainly evaluated by calculating four distances, including weighted_unifrac, unweighted_unifrac, Jaccard, and bray_curtis. All the above indicators were calculated by QIIME2. The analysis of differences between groups based on the obtained species abundance was performed using the Kruskal-Wallis test. The Mann–Whitney test or Kruskal-Wallis ANOVA was used to compare other discrete data. The Wilcoxon signed-rank test was used for paired comparison of continuous parameters. Bar plots, Receiver operating characteristic curve (ROC) curves, and area under the ROC curve (AUC) values were all generated in R.

## Results

### Subject clinical characteristics and sample information

A total of 120 subjects were recruited for the study. All had unilateral pulmonary nodules on chest X-rays and had not been treated with any antibiotics within 1 month. The BALF samples were prospectively collected from all patients for flora diversity analysis. Eleven patients diagnosed by histopathology as non-lung primary tumors (from metastases) were excluded, and the remaining 109 BALF samples were subjected to 16S rRNA gene sequencing. In order to eliminate the potential contamination of the bronchoscope, the lumen was flushed with 10 ~ 20 ml of sterile saline before bronchoscopy in 10 randomly selected subjects. The flushing fluid was collected with sterile centrifuge tubes and used as negative control samples for 16S rRNA sequencing.

Twenty-three BALF samples were excluded because they did not meet the minimum sequencing depth requirement (<1,000 reads/sample). Ultimately, 90 subjects [70 lung cancer patients (Tumor group) and 20 benign nodule patients (Non-tumor group)] and six negative control samples were included in the analysis, and the detailed sample filtering process is described in Supplementary Figure S1. The 70 lung cancer patients included 31 LUADs, 22 lung squamous carcinomas (LUSCs), and 17 small cell lung cancers (SCLCs). The demographic and clinical data of the patients are shown in Table 1.

**Table 1:** Demographic and clinical characteristics of the cohort.

	Non-Tumor (*n* = 20)	Tumor				*p* value (Non-Tumor *vs* Merged)
		LUAD (*n* = 31)	SCLC (*n* = 17)	LUSC (*n* = 22)	Merged (*n* = 70)	
Age, year						0.792^1^
Mean ± SD	58.50 ± 7.16	59.97 ± 6.50	58.71 ± 5.49	57.86 ± 6.14	59.00 ± 6.14	
Gender						0.201^2^
Female, *n* (%)	10 (50%)	18 (58.1%)	3 (17.6%)	3 (13.6%)	24 (34.3%)	
Male, *n* (%)	10 (50%)	13 (41.9%)	14 (82.4%)	19 (86.4%)	46 (65.7%)	
Smoking status						0.102^2^
Current/former	7 (35.0%)	8 (25.8%)	13 (76.5%)	18 (81.8%)	39 (55.7%)	
Never	13 (65.0%)	23 (74.2%)	4 (23.5%)	4 (18.2%)	31 (44.3%)	
AJCC 8th stages						
I	–	21 (67.7%)	13 (76.5%)	13 (51.9%)	47 (67.1%)	
II	–	6 (19.4%)	1 (5.9%)	6 (27.3%)	13 (18.6%)	
III	–	3 (9.7%)	2 (11.8%)	2 (9.1%)	7 (10.0%)	
IV	–	1 (3.2%)	1 (5.9%)	1 (4.5%)	3 (4.3%)	

^1^Mann–Whitney U test; ^2^Pearson’s chi-squared test. AJCC, American Joint Committee on Cancer; LUAD, lung adenocarcinoma; LUSC, lung squamous cell carcinoma; SCLC, small cell lung cancer. *n*, represents the number of patients.

### Background flora of bronchoscope

Potential background bronchoscopic flora was observed prior to subject sample analysis. This detected DNA is often thought to be from inanimate organisms or free DNA fragments, and ignoring their presence may bias the analysis results. The result is that even if we use the maximum loading volume of 3 ul of DNA template allowed by the instrument, only six genomic DNA samples at the risky level were finally obtained, which indicates that the genomic DNA content of the negative control samples is very low. As shown in Supplementary Figures S2A,B, the number of amplicon sequence variants (ASVs) obtained was below 50 when the sequencing depth was sufficient. Principal component analysis (PCA) analysis showed a relatively concentrated distribution of the six negative control samples (Supplementary Figure S2C). This result suggests that the negative control samples randomly collected in our existing working environment had a relatively stable background flora. At the phylum, order, family, and genus levels, all six samples had a similar community structure (Supplementary Figures S2D–G), with Pseudomonas and Rhodococcus predominating at the genus level. Subsequent analysis of the subject samples showed a sparse representation of these two genera (414/819 and 532/819, respectively, when sorted by abundance) and no significant difference between the Non-tumor group and Tumor group. These results suggest that these background bacteria do not interfere with the analysis results.

### Bacterial diversity of subjects in non-tumor and tumor groups

**Figure 1:**
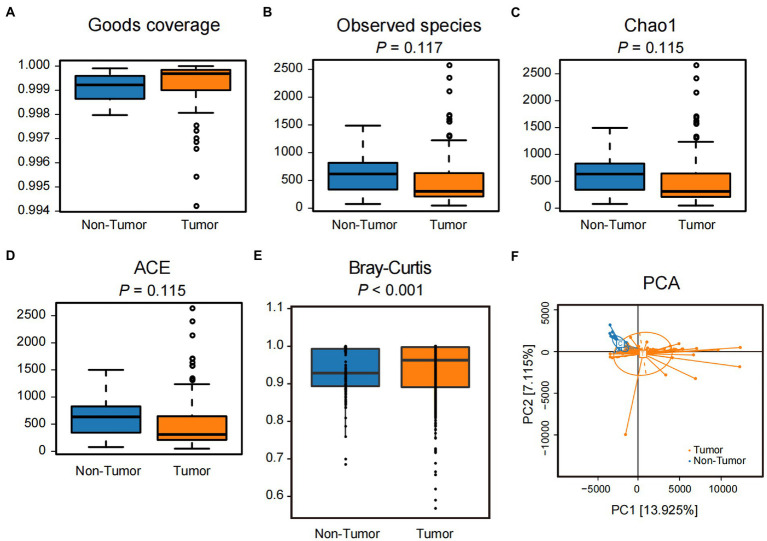
Difference analysis of *α* and *β* diversities of BALF samples in Tumor and Non-tumor groups. **(A)** Goods coverage refers to the coverage of the two sample libraries, whose value is close to 1, indicating that the sequencing depth is sufficient. **(B)** Observed species, number of observed ASVs. **(C)** Chao1 index, one of the *α* diversity indicators to measure community richness. The larger the value, the more taxa. **(D)** ACE index, another *α* diversity indicator to measure community richness, differs from the algorithm for the Chao1 index. **(E)** Bray-Curtis distance, one of the quantitative indicators of *β* diversity among samples. **(F)** PCA analysis based on ASV abundance and Euclidean distance showed a scattered distribution of samples between the two groups.

The goods coverage values of both groups were greater than 0.999, indicating that the sequencing depth covered the majority of taxa in the subject samples (Figure 1A). The actual number of ASVs observed in the Tumor group was slightly lower than in the Non-tumor group but not statistically different (*p* = 0.117; Figure 1B). The number of ASVs in the communities estimated by Chao1 and ACE indices in the Tumor group was also lower than that in the Non-tumor group, and there was no significant difference (*p* = 0.115; Figures 1C,D). There was no significant difference in *α* diversity between the two groups estimated by Simpson and Shannon indices (*p* > 0.05; Supplementary Figures S3A,B). The Non-tumor group was compared with the three pathological subtypes (LUAD, LUSC, and SCLC) separately, and the results also showed no significant difference in *α* diversity (*p* > 0.05; Supplementary Figures S4A–F).

Quantitative analysis of the differences in community structure between the two groups based on Bray-Curtis distance showed that the Tumor group had a significantly higher *β* value than the Non-tumor group (*p* < 0.001; Figure 1E). Weighted and unweighted UniFrac distance analysis also showed that the *β* value of the Tumor group was significantly higher than that of the Non-tumor group (*p* < 0.05; Supplementary Figures S3C,D). PCA analysis based on Euclidean distance showed a dispersed distribution between the Tumor and Non-tumor groups (Figure 1F). Similarly, Bray-Curtis distance-based principal coordinate analysis (PCoA) analysis and UniFrac distances-based nonmetric multidimensional scaling (NMDS) analysis could clearly distinguish the differences between the two groups (Supplementary Figures S3E,F). Inter-subgroup comparisons likewise showed that *β* values were higher in LUAD, LUSC, and SCLC than in the Non-tumor group (*p* < 0.05, Supplementary Figures S4G–I), whereas the differences between the three subgroups were minor. Three pathological subtypes were significantly separated from the Non-tumor group (Supplementary Figures S4J–L). Overall, these results suggest that the Tumor group, which includes all three pathological subtypes, showed no significant difference in *α* diversity and a significant increase in *β* diversity compared to the Non-tumor group.

### Different bacterial communities in non-tumor and tumor groups

**Figure 2:**
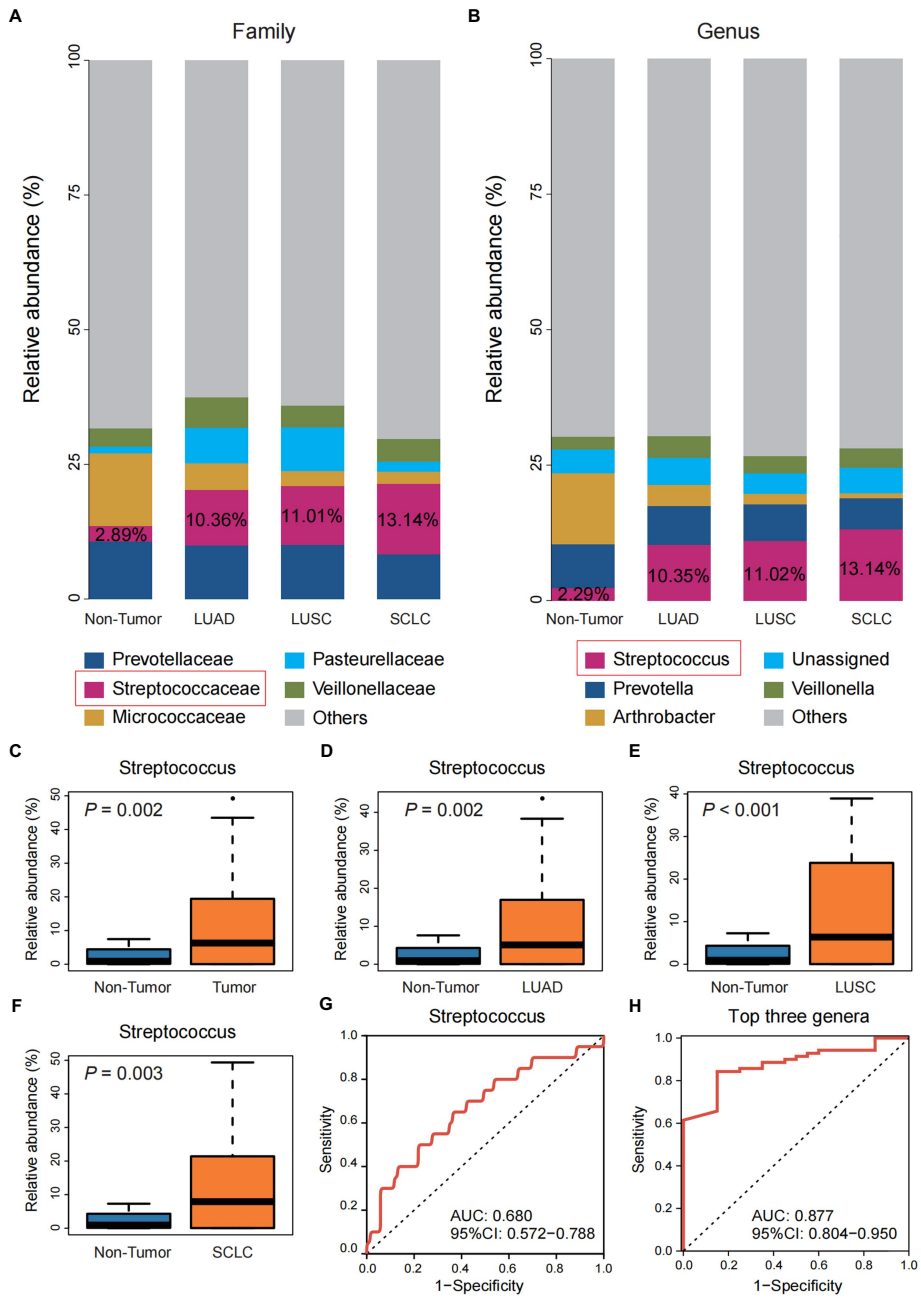
Differential analysis of the community structure of Tumor and Non-tumor groups. **(A)** The relative abundance of three pathological subtypes of lung cancer and the Non-tumor group at the family level. **(B)** The relative abundance of three pathological subtypes of lung cancer and the Non-tumor group at the genus level. **(C–F)** Comparison of the relative abundance of Streptococcus in group Non-tumor group with the overall Tumor group and the three pathological subtypes, respectively. **(G)** Receiver operating characteristic (ROC) curve with genus Streptococcus. **(H)** ROC curve with the top three genera, including Streptococcus, Prevotella, and Arthrobacter.

Compared with the Non-tumor group, family-level taxa abundance analysis showed that Streptococcaceae was significantly enriched in the three pathological subtypes of lung cancer, and the enrichment trend of Streptococcus was more pronounced at the genus level (Figures 2A,B). Difference analysis showed that the abundance of Streptococcus was significantly higher in the total Tumor group and the three pathological subtypes than in the Non-tumor group (*p* < 0.01; Figures 2C–F). ROC analysis showed that the ROC value of individual Streptococcus was 0.680 (Figure 2G), which was consistent with the AUC value of Streptococcus was 0.693 reported by Liu et al., (2018) in the protected specimen brushing (PSB) samples. The combination of the top three genera, Streptococcus, Prevotella, and Arthrobacter, showed a higher ROC value (0.877) and stronger predictive power for lung cancer than Streptococcus alone (Figure 2H).

### *Streptococcus pneumoniae* promotes the migration and invasion of A549 cells and H1299 cells *in vitro*

Although the ability of 16S rRNA sequencing to further differentiate Streptococcus at the species level is very limited, *S. pneumoniae* with typical pathogenic characteristics appealed to us. First, the potential role of virulent *S. pneumoniae* in lung cancer progression has not been sufficiently appreciated compared to commensal streptococci such as *Streptococcus mitis* (*S. mitis*); Second, a large population-based cohort study has found that the incidence of lung cancer in patients with pneumococcal pneumonia is significantly higher than that in patients without a history of pneumococcal pneumonia (RR: 3.25; 95% CI: 3.09–3.42; *p* < 0.001; Lin et al., 2014); Third, we detected *S. pneumoniae* in 21.8% (12/55) of lung cancer tissue samples in two independent laboratories (Jinan and Qingdao, China; Supplementary Figure S5).

**Figure 3:**
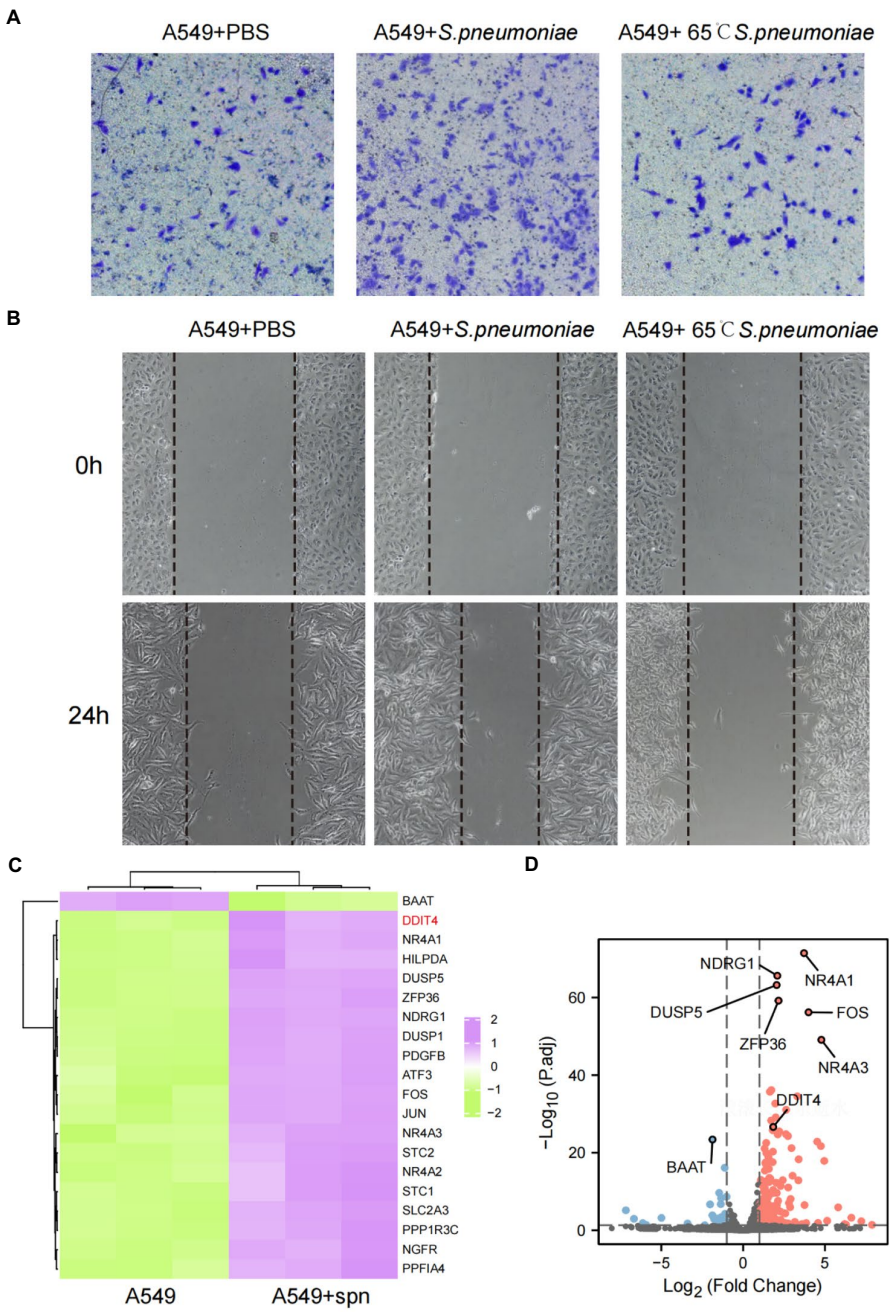
Enhanced migration and invasive abilities of A549 cells and transcriptome changes after infection with *S. pneumoniae*. **(A)** Transwell migration assays of A549 cells co-cultured with PBS, live *S. pneumoniae*, and heat-killed *S. pneumoniae*, respectively. **(B)** Cell scratch assays of A549 cells co-cultured with PBS, live *S. pneumoniae*, and heat-killed *S. pneumoniae* for 0 and 24 h, respectively. **(C)** Compared with the control group, the top 20 mRNAs with the most significant changes after co-culture of A549 cells with *S. pneumoniae*. **(D)** Volcano plot of significantly different mRNAs.

To assess the effect of *S. pneumoniae* infection on the migration and invasion of lung cancer cells, we performed cell scratch and transwell assays after co-culturing A549 cells with *S. pneumoniae* for 4 h. The results showed that viable *S. pneumoniae* infection significantly enhanced the migration rate and invasion of A549 cells, while heat-killed *S. pneumoniae* did not have this biological effect (Figures 3A,B; Supplementary Figure S6). It is suggested that this pro-migration and invasion effect may depend on the integrity of pneumococcal membrane proteins. Furthermore, adding oxacillin to the co-culture system to kill *S.pneumoniae* significantly weakened the biological effect (Supplementary Figure S7).

H1299 is another LUAD cell line with a unique p53 protein deficiency profile. The results also showed that viable *S. pneumoniae* infection significantly enhanced the migration rate and invasion of H1299 cells (Supplementary Figure S8).

### *Streptococcus pneumoniae* infection promotes high expression of DDIT4 in A549 cells and significantly affects patient prognosis

**Figure 4:**
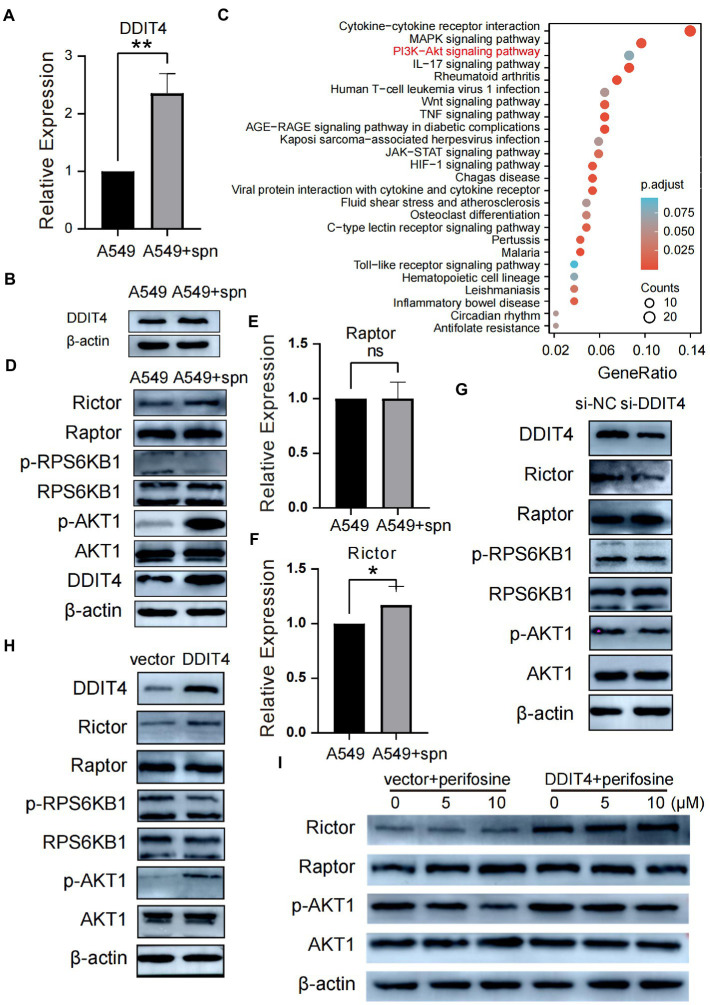
Up-regulated DDIT4 regulates the mTORC2/AKT signaling pathway. **(A)** Differential expression of the DDIT4 gene in the co-culture and A549 control groups was analyzed using qPCR, **P < 0.01. **(B)** The differential expression of DDIT4 protein in the co-culture and A549 control groups was analyzed by WB. **(C)** KEGG enrichment analysis based on significantly differentially expressed genes between the co-culture group and the A549 control group. **(D)** Compared with the control group, the expression of Rictor and phosphorylated AKT (p-AKT) in A549 cells in the co-culture group was up-regulated. At the same time, the remaining proteins had no significant change. **(E,F)** qPCR similarly confirmed that the Rictor gene but not the Raptor gene was up-regulated in the co-culture group, *P < 0.05. **(G)** Silencing of the DDIT4 gene resulted in reduced expression of Rictor and p-AKT without significant effects on other proteins. **(H,I)** Overexpression of DDIT4 up-regulated the expression of Rictor and p-AKT1, which was inhibited by the addition of AKT inhibitor perifosine.

To explore the underlying molecular mechanism by which *S. pneumoniae* infection enhances the migratory and invasive of A549 cells, we performed mRNA sequencing of A549 cells co-cultured with *S. pneumoniae* for 4 h. The results showed that the expression of multiple mRNAs was altered in the co-culture group of A549 cells compared with the PBS control group (NC), including 632 up-regulated mRNAs and 158 down-regulated mRNAs (*p* < 0.05, |log2 (foldchange)| > 1; Supplementary Table S1). Among these differentially expressed mRNA, DDIT4, also known as regulated in DNA damage and development 1 (REDD1) or HIF-1 Responsive Protein RTP801, is particularly prominent (Figures 3C,D). It is one of the most abundantly expressed mRNAs, and the most significantly differentially expressed mRNAs between the two groups (*p* = 2.05E-30, log2 (fold change) = 1.84). WB and qPCR confirmed that *S. pneumoniae* infection did increase the expression level of DDIT4 in A549 cells (Figures 4A,B).

We further analyzed DDIT4 using public databases on the TissGDB website,^1^ the tissue-specific gene annotation database in cancer. The results showed that DDIT4 was expressed at low levels in most normal mature tissues, up-regulated in a variety of malignancies, including LUAD, and only one isoform was found (Supplementary Figures S9A–C). High expression of DDIT4 is associated with overall survival (OS) and relapse-free survival (RFS) shortening in most tumor types (Supplementary Figure S9D). In patients with LUAD, high expression of DDIT is an unfavorable prognostic factor (Supplementary Figure S9E). ROC curve analysis showed that the AUC of DDIT4 in total lung cancer, LUAD and LUSC was about 0.6 (Supplementary Figure S9F), suggesting that DDIT4 alone has a limited ability to predict the prognosis of lung cancer patients and needs to be analyzed together with other indicators.

### *Streptococcus pneumoniae* regulates mTORC2/AKT signal pathway by up-regulating DDIT4 expression

Kyoto Encyclopedia of Genes and Genomes (KEGG) analysis based on mRNA sequencing showed that IL-17, Wnt, MAPK, NF-κB, JAK–STAT, PI3K-Akt, and other pathways were up-regulated by *S. pneumoniae* infection (Figure 4C). Previous studies have shown that conditions such as hypoxia and stress can up-regulate DDIT4 expression (Shoshani et al., 2002; Cho et al., 2018), but notably, the role of DDIT4 in tumors appears to be paradoxical and tumor environment-dependent. DDIT4 can act as a tumor suppressor in colorectal cancer (Wang et al., 2015), breast cancer (Horak et al., 2010), and non-small cell lung cancer (NSCLC; Jin et al., 2011) by inhibiting mTORC1, and can also play a pro-cancer role in ovarian cancer (Chang et al., 2018), bladder urothelial carcinoma (Zeng et al., 2018) and gastric cancer (Du et al., 2018) by reducing apoptosis. The current report on the interaction of DDIT4 and Akt is also ambiguous. Dennis et al. (2014) reported that DDIT4 mediated Akt dephosphorylation by enhancing protein phosphatase 2A, while Jia et al. (2014) reported a positive correlation between DDIT4 and p-Akt. How DDIT4 regulates the AKT pathway during up-regulation of A549 cell migration and invasion ability by *S. pneumoniae* infection is unclear; therefore, we aimed to explore the potential regulatory relationships among DDIT4, mTOR (including mTORC1 and mTORC2), and Akt to find specific pathways by which *S. pneumoniae* regulates the enhanced migration and invasion ability of A549 cells.

After co-culturing A549 cells with *S. pneumoniae* for 4 h, compared with the PBS control, WB results showed that the protein expression level of Raptor in the mTORC1 complex did not increase, and the protein expression level of RPS6KB1, which is downstream of mTORC1, did not change, and the protein level of phosphorylated RPS6KB1 (p-RPS6KB1) was also not increased (Figure 4D). The qPCR results also confirmed that the transcriptional level of Raptor did not increase (Figure 4E). These results suggest that the mTORC1 complex was not activated by increased DDIT4 expression induced by *S. pneumoniae*. In contrast, both qPCR and WB results showed increased transcription and expression levels of Rictor, a vital member of the mTORC2 complex (Figures 4D,F).

We then analyzed the expression of AKT1 protein, which can be a downstream target of mTORC2. WB results showed no significant change in AKT1 expression levels, but the expression level of the phosphorylated active form p-AKT1 was significantly increased (Figure 4D). To verify the interaction between DDIT4 and the AKT pathway, we performed DDIT4 loss- and gain-of-function experiments in A549 cells. WB results showed that silencing DDIT4 in A549 cells suppressed Rictor and p-AKT protein expression (Figure 4G), while over-expression of DDIT4 significantly up-regulated Rictor and p-AKT expression (Figure 4H). There was no significant change in the expression of Raptor, a molecule related to the mTORC1 pathway. The p-AKT protein expression level decreased with increasing inhibitor concentration after over-expression of DDIT4, followed by the addition of the AKT inhibitor Perifosine. There was no significant change in Raptor expression level (Figure 4I). These results suggest that *S. pneumoniae* infection activates the mTORC2/AKT signaling pathway through up-regulation of DDIT4 expression.

### DDIT4 promotes migration and invasion of A549 cells *in vitro* by regulating the AKT pathway

**Figure 5:**
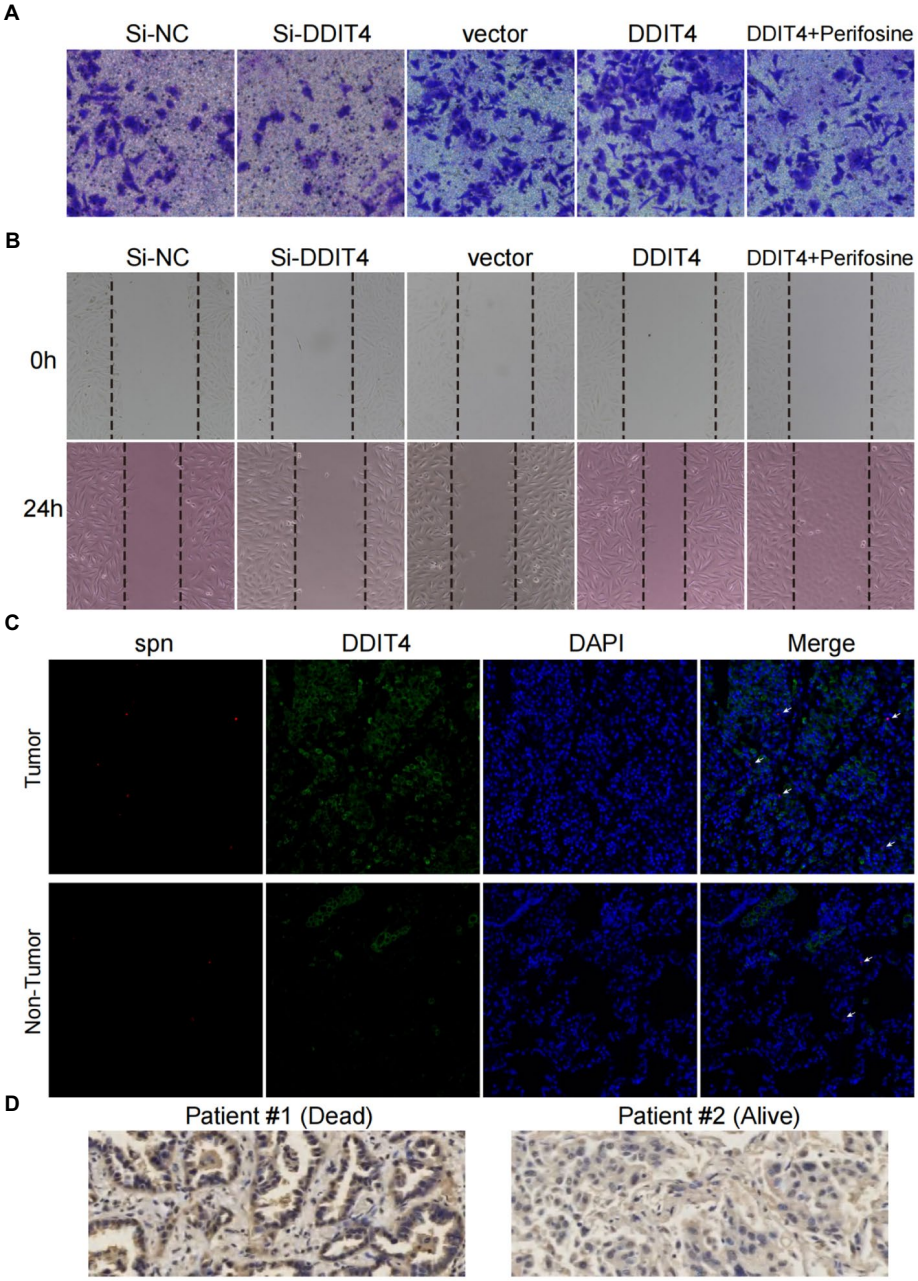
High expression of DDIT4 enhances the migratory and invasive abilities of A549 cells and impairs the prognosis of LUAD patients. **(A)** Transwell migration assays of A549 cells with silencing of DDIT4, overexpression of DDIT4, overexpression of DDIT4 followed by addition of AKT inhibitor perifosine, and blank control. **(B)** Cell scratch assays of A549 cells with silencing of DDIT4, overexpression of DDIT4, overexpression of DDIT4 followed by addition of AKT inhibitor perifosine, and blank control at 0 and 24 h, respectively. **(C)** Expression and enrichment of DDIT4 and *S. pneumoniae* in lung cancer tissues. The white arrows refer to visible *S. pneumoniae*. **(D)** Expression of DDIT4 protein detected by immunohistochemistry in LUAD patients with different prognoses.

Cell scratch and transwell assays were used to evaluate the effects of loss- and gain-of-function of DDIT4 and AKT on the migration and invasion of A549 cells *in vitro*. Silencing DDIT4 expression in A549 cells significantly inhibited cell migration, and over-expression of DDIT4 promoted cell migration, but its pro-migration ability was significantly counteracted by the addition of an AKT inhibitor (Figure 5A; Supplementary Figures S10A,B). Similarly, the same trend was observed in the cell scratch experiment (Figure 5B; Supplementary Figures S10C,D). These results confirm that DDIT4 can promote the migration and invasion of A549 cells through up-regulation of the AKT pathway.

**Figure 6:**
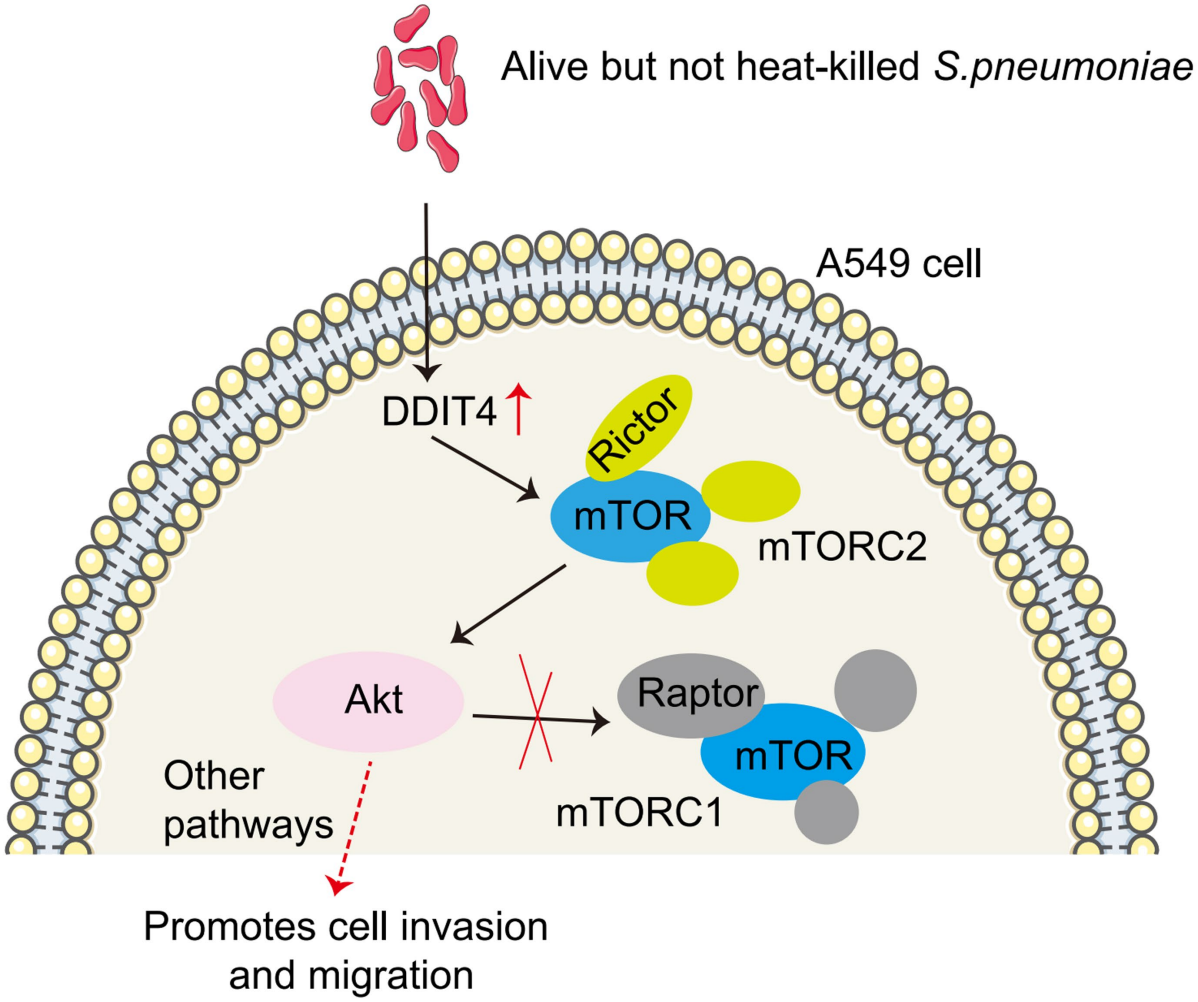
Model of *S. pneumoniae* up-regulates DDIT4 expression in A549 cells, which activates the mTORC2/AKT signaling pathway.

### Enrichment or over-expression of *Streptococcus pneumoniae* and DDIT4 in LUAD tissues

To determine the clinical significance of *S. pneumoniae* and DDIT4, we visualized the expression of DDIT4 and the enrichment of *S. pneumoniae* in LUAD tissues using IF and FISH. The results showed that the expression of DDIT4 in LUAD tissues was significantly higher than that in paracancerous tissues, and *S. pneumoniae* was significantly more enriched in LUAD tissues. Moreover, high enrichment of *S. pneumoniae* and over-expression of DDIT4 could be observed in the same region of LUAD tissues (*p* = 0.034; Figure 5C; Supplementary Figure S11). Finally, we randomly selected a LUAD patient with a good prognosis and a LUAD patient who died due to multiple metastases by follow-up. The IHC results also showed that DDIT4 was significantly highly expressed in this patient with a poor prognosis (Figure 5D).

## Discussion

In the present study, we observed that compared with patients with benign pulmonary nodules, the *α* diversity of BALF in patients with lung cancer tended to decrease. Still, there was no significant difference, consistent with the results observed by Kovaleva et al. in 26 pairs of NSCLC tissues and paired adjacent tissues. In contrast, *β* diversity was significantly higher in patients with lung cancer and had consistent results in different histological types. Notably, there are conflicting results in the current reports of altered *α* diversity and *β* diversity of flora in lung cancer (Morton and Singanayagam, 2022). These controversial findings may result from significant differences in study populations, specimen sources, and sampling methods.

Among the changes in abundance at the family and genus level, *Streptococcaceae* and *Streptococcus* were significantly more abundant. They had the most significant proportion of lung cancer patients compared to patients with benign lung nodules, and the same results were observed in LUAD, LUSC, and SCLC. Liu et al. (2018) also observed a significant increase in Streptococcus in PSB samples collected from 24 Chinese patients with unilateral lung cancer compared to 18 healthy controls. In this study, ROC analysis showed that the AUC of Streptococcus in BALF was 0.680, which had limited predictive ability for lung cancer, similar to the AUC value of 0.693 observed by Liu et al. (2018) in PSB samples. In contrast, Bello et al. (2021) observed an AUC of 0.897 for Streptococcus in bronchial biopsies from 25 Spanish patients with central lung cancer and 16 controls. It is worth mentioning that when we combined the three genera Streptococcus, Prevotella, and Arthrobacter with the most significant proportions at the genus level, the AUC was 0.877, which has good predictive power, indicating that the combination of genera can better predict lung cancer to a certain extent.

Streptococcus dysregulation is associated with the occurrence and progression of various tumors, including lung cancer. Ma et al. (2017) found that *Streptococcus salivarius* significantly increased levels of Th17 cells, and Th17 cell-mediated inflammation in the lung has been identified as an important factor in promoting the occurrence and metastasis of lung cancer (Chang et al., 2014). In this study, we found that *S. pneumoniae* infection increased the migration and invasion of A549 cells *in vitro* and led to the up-regulation of Wnt, MAPK, Akt, and other signal pathways. Interestingly, Tsay et al. (2018) also found an enrichment of Streptococcus in the airways of lung cancer and that exposure of A549 cells to *S. mitis* resulted in up-regulation of ERK/MAPK and PI3K/Akt signaling pathways.

The origin of these intratumoral bacteria, including *S. pneumoniae*, and whether they are a cause or a consequence of the tumor process need to be addressed. The oral cavity is traditionally considered a natural habitat for streptococci, and the oral/lung bacterial exchange could occur *via* microaspirations (Segal et al., 2016; Tsay et al., 2021). It was initially thought that the lower airway microbiome represented contamination by microbiomes in the upper airway or oral cavity. However, similar results were obtained from several subsequent studies, indicating that the possibility of pollution is minimal (Huang et al., 2013). It is worth noting that most of the current reports, including our study, are cross-sectional study designs that cannot determine a causal relationship between the microbiome and lung cancer but only observe the fluctuations in infection load or bacterial composition ratios during disease progression (Goto, 2020). Nevertheless, the microbiome is attracting attention as a biomarker of cancer development because they are located in the vicinity of tumor tissue and may invade peritumor tissues.

Some studies have also reported that Staphylococcus can induce DNA damage, while Streptococcus can prevent it and exert an anticancer effect (Urbaniak et al., 2016), although (Hosgood et al., 2014; Liu et al., 2018) reported the opposite results, respectively. These contradictory results may arise from differences in the tumor microenvironment, and another possible explanation is the need to identify the actual species or strains involved in carcinogenesis. Most current studies have focused on commensal streptococci traditionally considered mild, such as *S. mitis*, *Streptococcus anginosus*, and *Streptococcus infantis*. In contrast, *S. pneumoniae*, as a typical pathogen, has not been fully explored. In this study, we found that *S. pneumoniae* co-cultured with A549 cells could enhance its migration and invasion ability and significantly up-regulated the expression of DDIT4, which activated mTORC2 and consequently up-regulated the Akt pathway. Jin et al. (2013) also observed in human LUAD cells H1299 that constitutive overexpression of DDIT4 could induce Akt activation, and selective inhibition of Akt and mTORC1/2 using Perifosine and PP242 effectively inhibited Akt phosphorylation induced by sustained over-expression of DDIT4 and increased cell sensitivity to cisplatin. High expression of DDIT4 has also been shown to be associated with poorer prognosis in patients with LUAD and is an independent predictor of OS (Song et al., 2021).

At the end of this study, we used IF combined with FISH to detect the expression of DDIT4 and the enrichment of *S. pneumoniae* in lung cancer tissues. The results showed that the expression of DDIT4 and the enrichment of *S. pneumoniae* were significantly higher in lung cancer tissues than in paracancerous tissues. Significantly different expression of DDIT4 was also confirmed in two LUAD patients with different prognoses, and patients with high DDIT4 expression had a poorer prognosis.

In conclusion, our study suggests that *S. pneumoniae* enrichment up-regulated DDIT4 expression in A549 cells, which promotes the migration and invasion of A549 cells through the mTORC2/AKT signaling pathway (Figure 6). This study provides a potential new mechanism for targeted therapy of lung cancer. However, several limitations must be addressed in future studies. First, our study was a single-center, cross-sectional study involving a small sample size, particularly in the benign pulmonary nodule group. Second, although we identified a correlation between *S. pneumoniae* and lung cancer, we did not provide potentially more comprehensive information. We observed that enriched *S. pneumoniae* promoted the migration and invasion of LUAD cells *in vitro*, but whether reduced Micrococcaceae and Arthrobacter may have opposite effects is unknown. Parallel testing of *S. pneumoniae*, *Prevotella melaninogenica*, *S. mitis*, and *Veillonella parvula* will help explain which bacteria possess this biological effect. Third, we found that *S. pneumoniae* infection enhanced DDIT4 expression and promoted the migration and invasion of lung cancer cells *in vitro* by up-regulating the AKT pathway. Our current test subjects are all transformed LUAD cell lines (A549 and H1299), and we have not yet observed the effect of *S. pneumoniae* on lung primary cells. Fourth, we did not validate this finding *in vivo* by establishing an animal model, nor did we observe whether the use of antibiotics to clear *S. pneumoniae* prevented progression in patients with early-stage lung cancer. Therefore, a larger sample size and multicenter studies in conjunction with metabolomics are needed to explore potential associations and molecular mechanisms between lower airway flora and lung cancer.

## Data availability statement

The datasets presented in this study can be found in online repositories. The names of the repository/repositories and accession number(s) can be found at: https://bigd.big.ac.cn/gsa/browse/CRA007616, CRA007616.

## Ethics statement

The studies involving human participants were reviewed and approved by the ethics committee of Qilu Hospital of Shandong University (Qingdao). The patients/participants provided their written informed consent to participate in this study.

## Author contributions

XS, GZ, and XP contributed to sample acquisition, data analysis, and manuscript preparation. BL and MD contributed to study conduct and data analysis. YX, BL, and XS contributed to study design, data interpretation, and manuscript revision. All authors contributed to the article and approved the submitted version.

## Funding

This work was supported by the Shandong Province medical staff science and technology innovation Plan project (SDYWZGKCJH2022014) and the Scientific Research Start-up Foundation of Shandong University Qilu Hospital (Qingdao) (QDKY2019ZD01).

## Conflict of interest

The authors declare that the research was conducted in the absence of any commercial or financial relationships that could be construed as a potential conflict of interest.

## Publisher’s note

All claims expressed in this article are solely those of the authors and do not necessarily represent those of their affiliated organizations, or those of the publisher, the editors and the reviewers. Any product that may be evaluated in this article, or claim that may be made by its manufacturer, is not guaranteed or endorsed by the publisher.

## Footnotes

^1^ https://bioinfo.uth.edu/TissGDB
