# Supplementary material for: Streptococcus pneumoniae promotes migration and invasion of A549 cells in vitro by activating mTORC2/AKT through up-regulation of DDIT4 expression
Source: Front Microbiol. 2022 Dec 19;13:1046226. doi: 10.3389/fmicb.2022.1046226 (PMC9806147; doi:10.3389/fmicb.2022.1046226)
Supplement: Supplementary file 1 [file Data_Sheet_1.PDF]

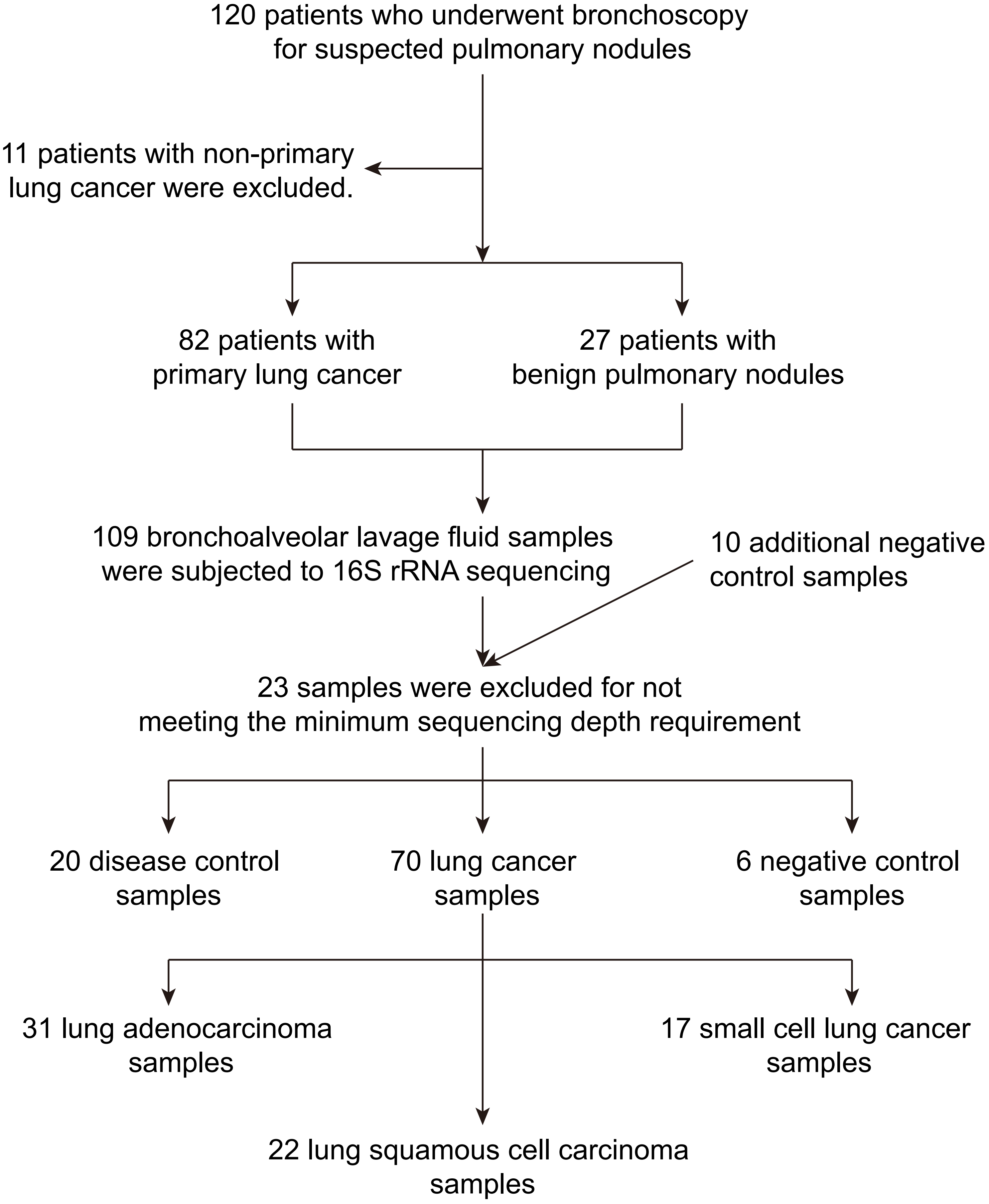
120 patients who underwent bronchoscopy for suspected pulmonary nodules
11 patients with non-primary lung cancer were excluded.
82 patients with primary lung cancer
27 patients with benign pulmonary nodules
109 bronchoalveolar lavage fluid samples were subjected to 16S rRNA sequencing
10 additional negative control samples
23 samples were excluded for not meeting the minimum sequencing depth requirement
20 disease control samples
70 lung cancer samples
6 negative control samples
31 lung adenocarcinoma samples
17 small cell lung cancer samples
22 lung squamous cell carcinoma samples

Figure S1. Workflow and sample filtering procedures.

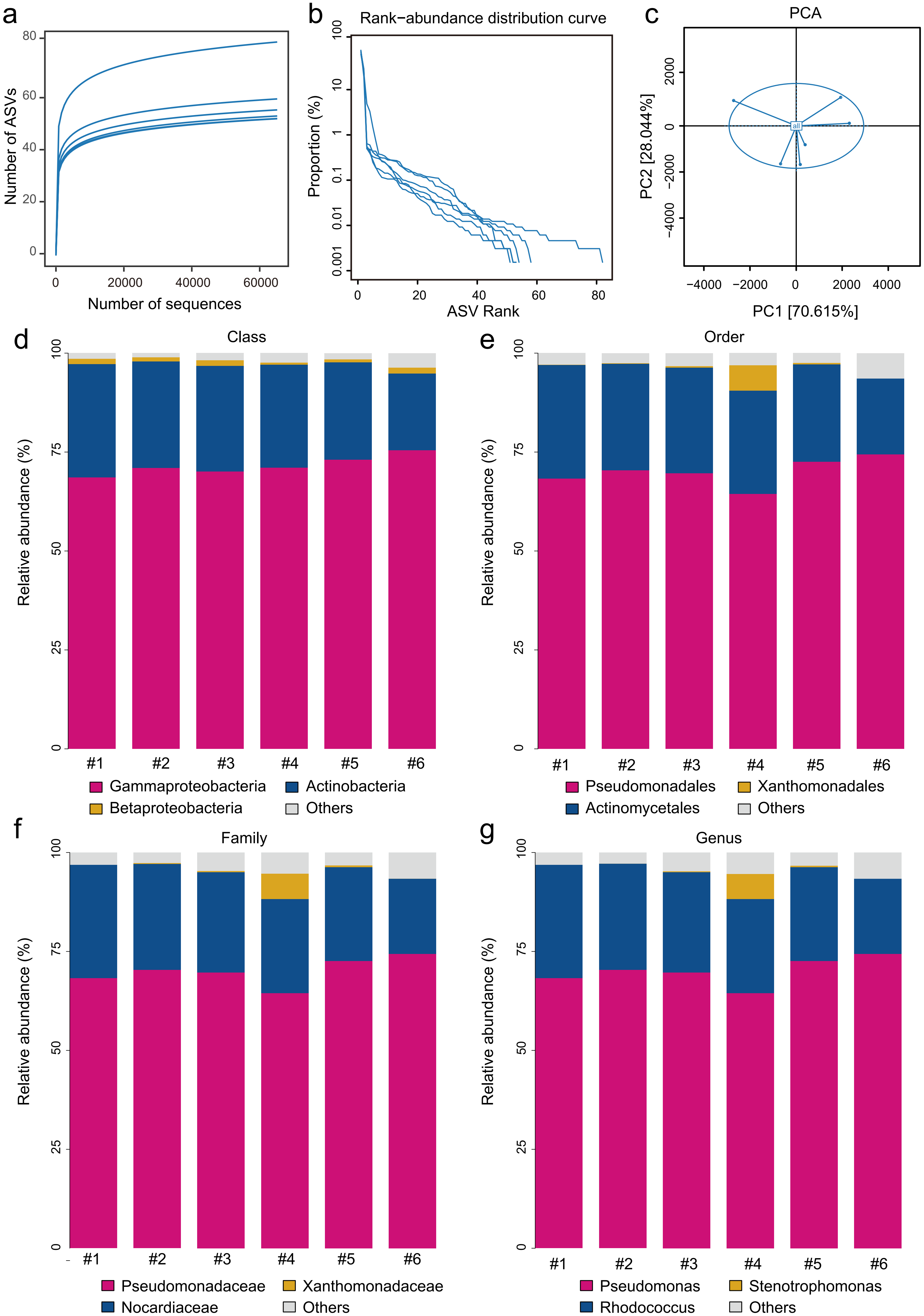
a
Number of ASVs
Number of sequences
b
Rank−abundance distribution curve
Proportion (%)
ASV Rank
c
PCA
PC2 [28.044%]
PC1 [70.615%]
all
d
Class
Relative abundance (%)
#1 #2 #3 #4 #5 #6
Gammaproteobacteria
Actinobacteria
Betaproteobacteria
Others
e
Order
Relative abundance (%)
#1 #2 #3 #4 #5 #6
Pseudomonadales
Xanthomonadales
Actinomycetales
Others
f
Family
Relative abundance (%)
#1 #2 #3 #4 #5 #6
Pseudomonadaceae
Xanthomonadaceae
Nocardiaceae
Others
g
Genus
Relative abundance (%)
#1 #2 #3 #4 #5 #6
Pseudomonas
Stenotrophomonas
Rhodococcus
Others

Figure S2. 16S rRNA sequencing results of 6 negative control samples. **(a)** The number of ASVs observed in the six samples. **(b)** Rank-abundance distribution curve, the curve's width reflects the community richness, and the smoothness of the curve reflects the community evenness. The distribution trends were consistent across the six samples. **(c)** PCA analysis also showed a relatively concentrated distribution of the six samples. **(d)** The relative taxa abundance of the six samples at the class level. **(e)** The relative taxa abundance of the six samples at the order level. **(f)** The relative taxa abundance of the six samples at the family level. **(g)** The relative taxa abundance of the six samples at the genus level.

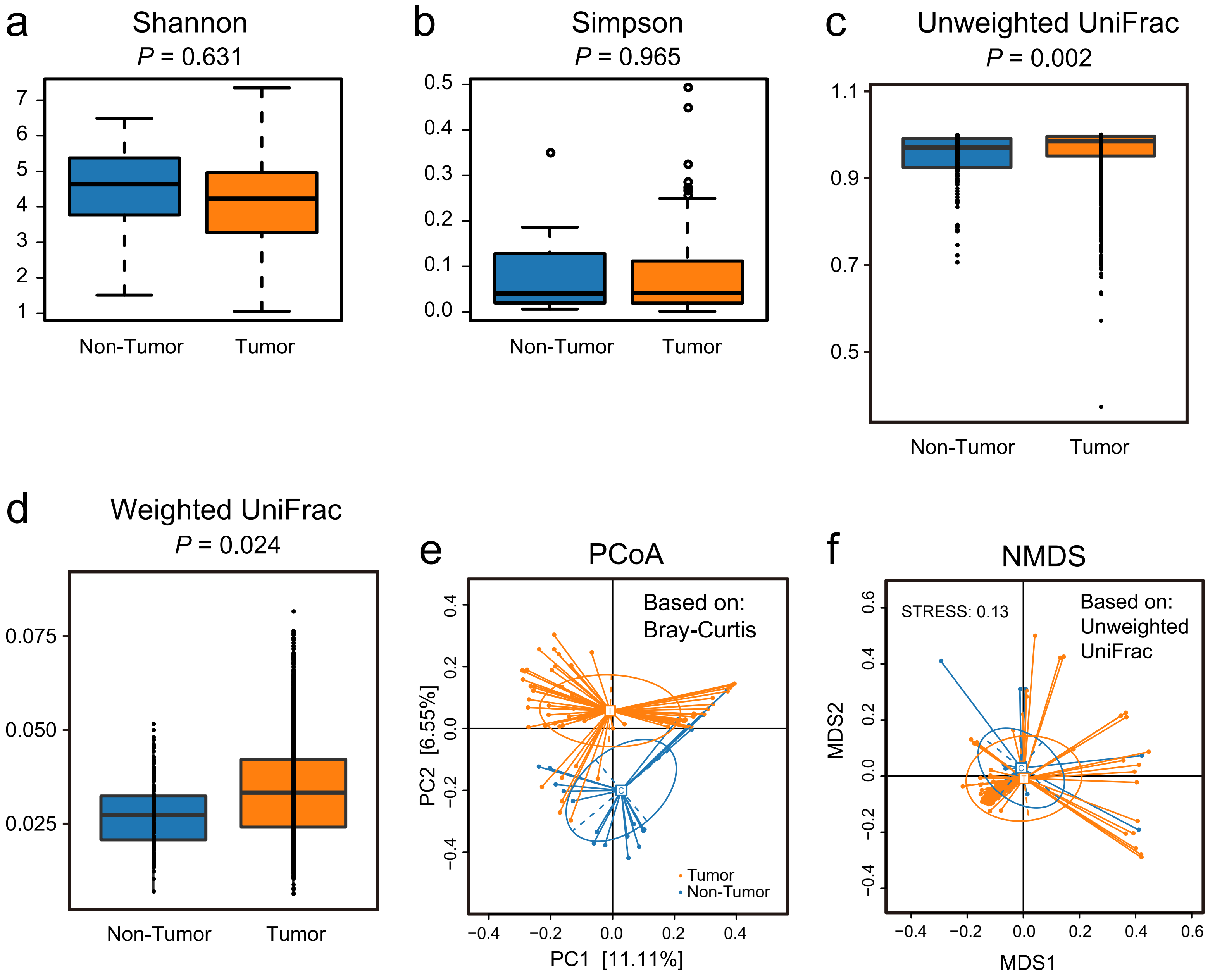

a
Shannon
P = 0.631
Non-Tumor
Tumor
b
Simpson
P = 0.965
Non-Tumor
Tumor
c
Unweighted UniFrac
P = 0.002
Non-Tumor
Tumor
d
Weighted UniFrac
P = 0.024
Non-Tumor
Tumor
e
PCoA
Based on: Bray-Curtis
Tumor
Non-Tumor
PC1 [11.11%]
PC2 [6.55%]
f
NMDS
STRESS: 0.13
Based on: Unweighted UniFrac
MDS1
MDS2

Figure S3. **(a)** Shannon index, one of the α diversity indices, the higher the value, the higher the community diversity. **(b)** Gini-Simpson index, another community diversity indicator. **(c)** Unweighted UniFrac distance, the qualitative measure of β diversity between samples based on the phylogenetic tree. **(d)** Weighted UniFrac distance, the quantitative measure of β diversity between samples based on the phylogenetic tree. **(e)** PCoA analysis based on Bray-Curtis distance, the results also showed that the samples were dispersed between the two groups. **(f)** NMDS analysis based on Jaccard distance; its stress value is 0.13.

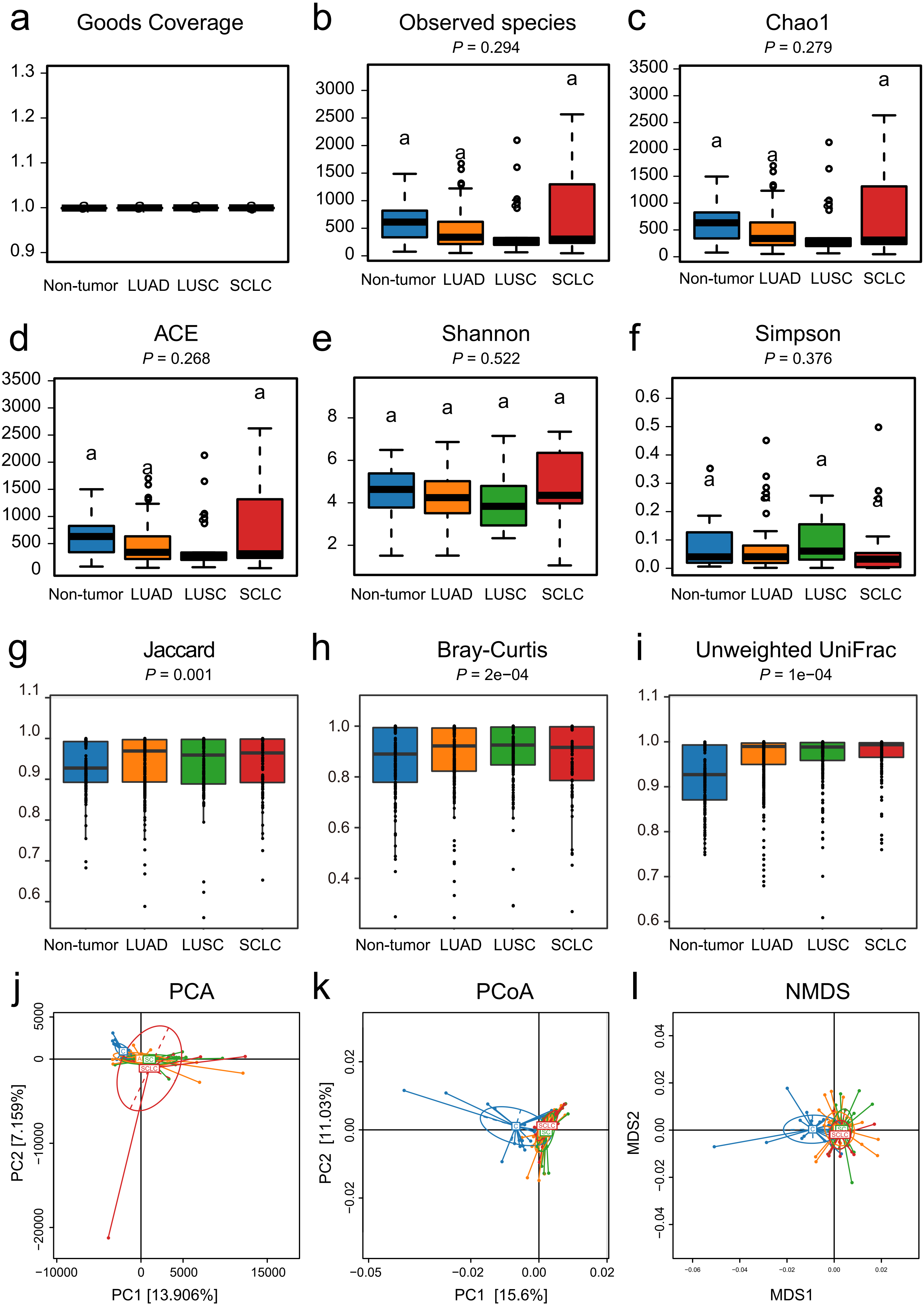
a
Goods Coverage
Non-tumor LUAD LUSC SCLC
b
Observed species
P = 0.294
Non-tumor LUAD LUSC SCLC
c
Chao1
P = 0.279
Non-tumor LUAD LUSC SCLC
d
ACE
P = 0.268
Non-tumor LUAD LUSC SCLC
e
Shannon
P = 0.522
Non-tumor LUAD LUSC SCLC
f
Simpson
P = 0.376
Non-tumor LUAD LUSC SCLC
g
Jaccard
P = 0.001
Non-tumor LUAD LUSC SCLC
h
Bray-Curtis
P = 2e−04
Non-tumor LUAD LUSC SCLC
i
Unweighted UniFrac
P = 1e−04
Non-tumor LUAD LUSC SCLC
j
PCA
PC1 [13.906%]
PC2 [7.159%]
k
PCoA
PC1 [15.6%]
PC2 [11.03%]
l
NMDS
MDS1
MDS2

Figure S4. Difference analysis of α and β diversities of BALF samples in the Non-tumor group and the three pathological subtypes. **(a)** Goods coverage values between the Non-tumor group and the three pathological subtypes. The results indicate that the sequencing depth is sufficient. **(b)** Observed species, number of observed ASVs. **(c-f)** Chao1 index, ACE index, Shannon index and Gini-Simpson index between the Non-tumor group and the three pathological subtypes. The results showed no significant differences in α diversity overall between the four groups. **(g-i)** Jaccard distance, Bray-Curtis distance and Unweighted UniFrac distance between the Non-tumor group and the three pathological subtypes. The results showed that the β diversity of all three pathological subtypes was higher than that of the Non-tumor group. **(j-l)** PCA, PCoA and NMDS all showed the three pathological subtypes clustered together, while separated from the Non-tumor group.

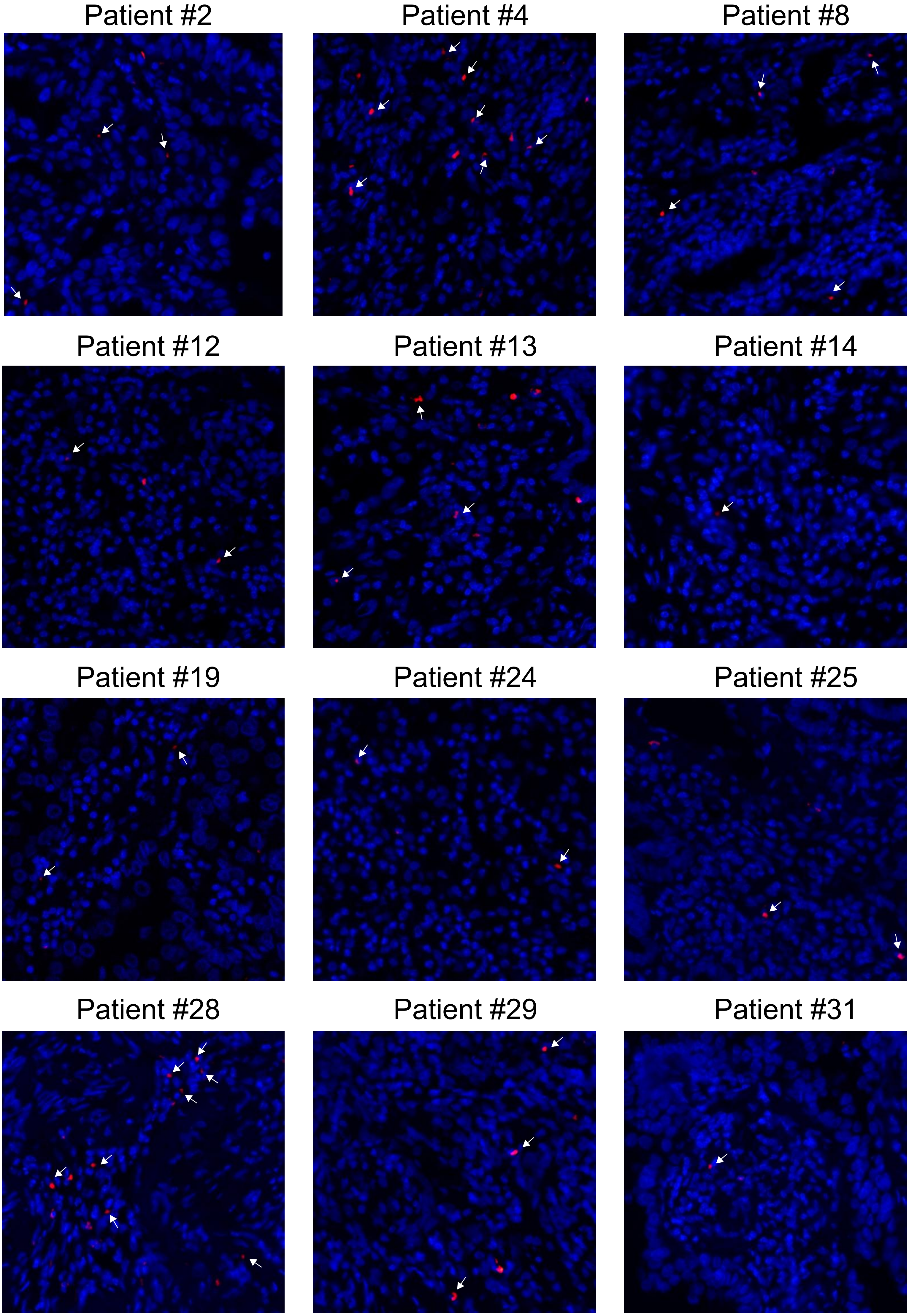
Patient #2
Patient #4
Patient #8
Patient #12
Patient #13
Patient #14
Patient #19
Patient #24
Patient #25
Patient #28
Patient #29
Patient #31

Figure S5. Tissue sections of 12 LUAD patients labeled with clearly visible *S. pneumoniae* using fluorescent in situ hybridization (FISH). The white arrow points to *S. pneumoniae*. Due to nuclear occlusion or layer hierarchy, we only labeled clear *S.pneumoniae*.

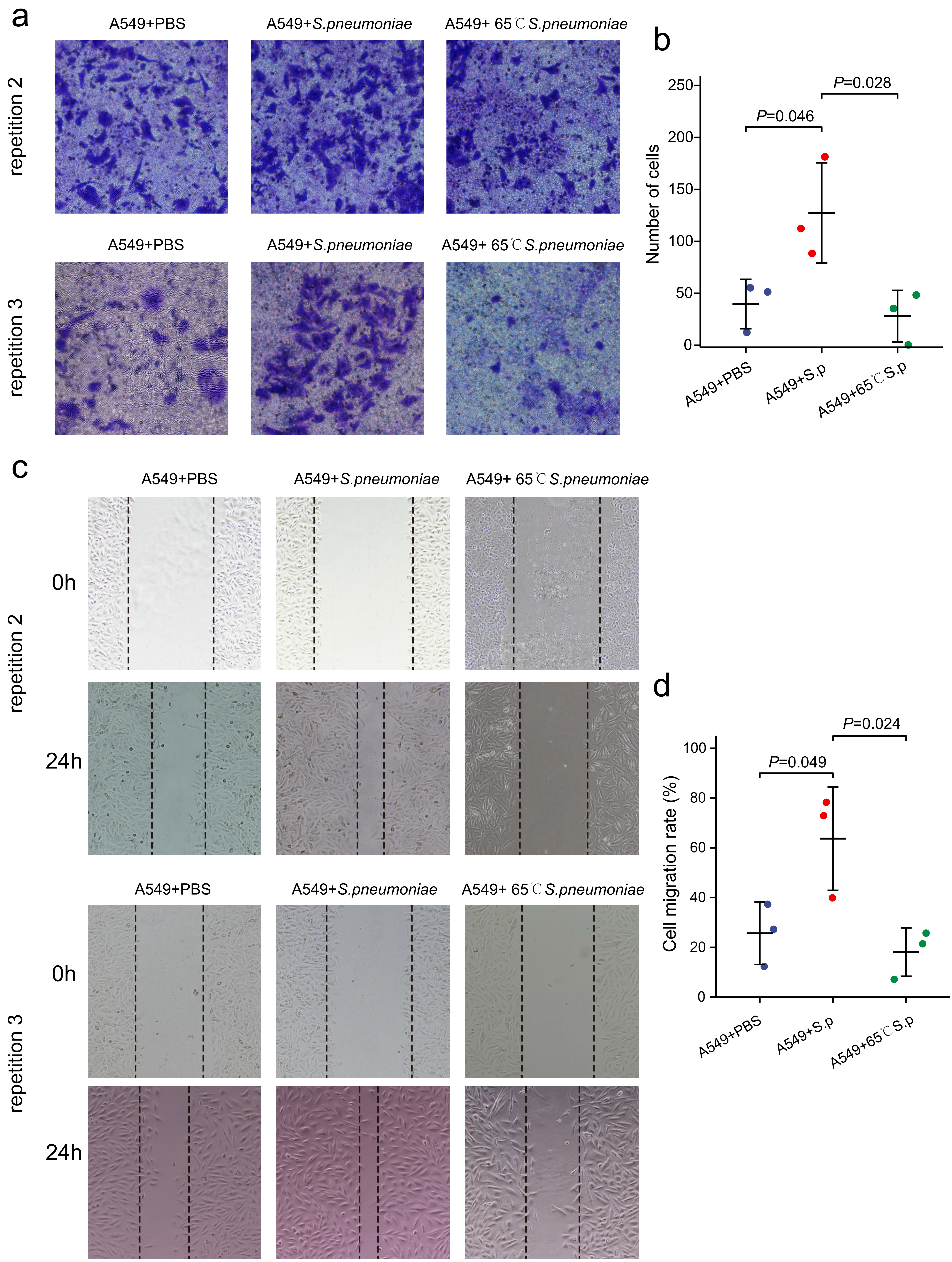

a
A549+PBS
A549+S.pneumoniae
A549+ 65℃S.pneumoniae
repetition 2
repetition 3
b
P=0.046
P=0.028
Number of cells
250
200
150
100
50
0
A549+PBS
A549+S.p
A549+65℃S.p
c
0h
24h
d
P=0.049
P=0.024
Cell migration rate (%)
100
80
60
40
20
0

Figure S6. Enhanced migration and invasive abilities of A549 cells after infection with *S. pneumoniae*. **(a)** Transwell migration assays of A549 cells co-cultured with PBS, live *S. pneumoniae*, and heat-killed *S. pneumoniae*, respectively. **(b)** One-way ANOVA and Tukey HSD Post hoc tests for the results of three experimental replications. **(c)** Cell scratch assays of A549 cells co-cultured with PBS, live *S. pneumoniae*, and heat-killed *S. pneumoniae* for 0 and 24 hours, respectively. **(d)** One-way ANOVA and Tukey HSD Post hoc tests for the results of three experimental replications.

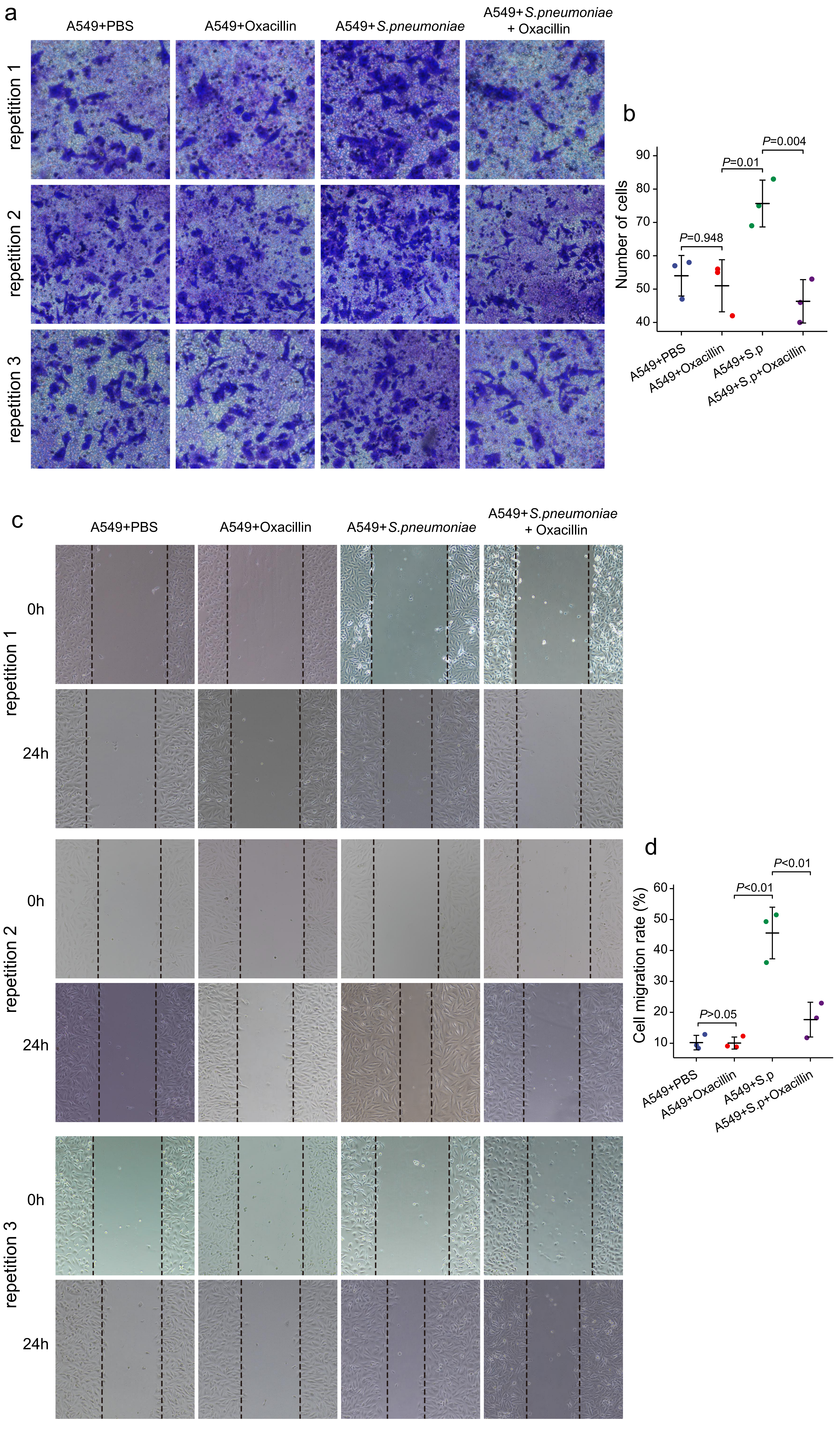
a
A549+PBS
A549+Oxacillin
A549+S.pneumoniae
A549+S.pneumoniae + Oxacillin
repetition 1
repetition 2
repetition 3
b
Number of cells
P=0.948
P=0.01
P=0.004
A549+PBS
A549+Oxacillin
A549+S.p
A549+S.p+Oxacillin
c
A549+PBS
A549+Oxacillin
A549+S.pneumoniae
A549+S.pneumoniae + Oxacillin
repetition 1
repetition 2
repetition 3
0h
24h
d
Cell migration rate (%)
P>0.05
P<0.01
P<0.01
A549+PBS
A549+Oxacillin
A549+S.p
A549+S.p+Oxacillin

Figure S7. Enhanced migration and invasive abilities of A549 cells promoted by *S. pneumoniae* was inhibited by the addition of oxacillin. **(a)** Transwell migration assays of A549 cells co-cultured with PBS, oxacillin, live *S. pneumoniae*, and both oxacillin and *S. pneumoniae*, respectively. **(b)** One-way ANOVA and Tukey HSD Post hoc tests for the results of three experimental replications. **(c)** Cell scratch assays of A549 cells co-cultured with PBS, oxacillin, live *S. pneumoniae*, and both oxacillin and *S. pneumoniae* for 0 and 24 hours, respectively. **(d)** One-way ANOVA and Tukey HSD Post hoc tests for the results of three experimental replications.

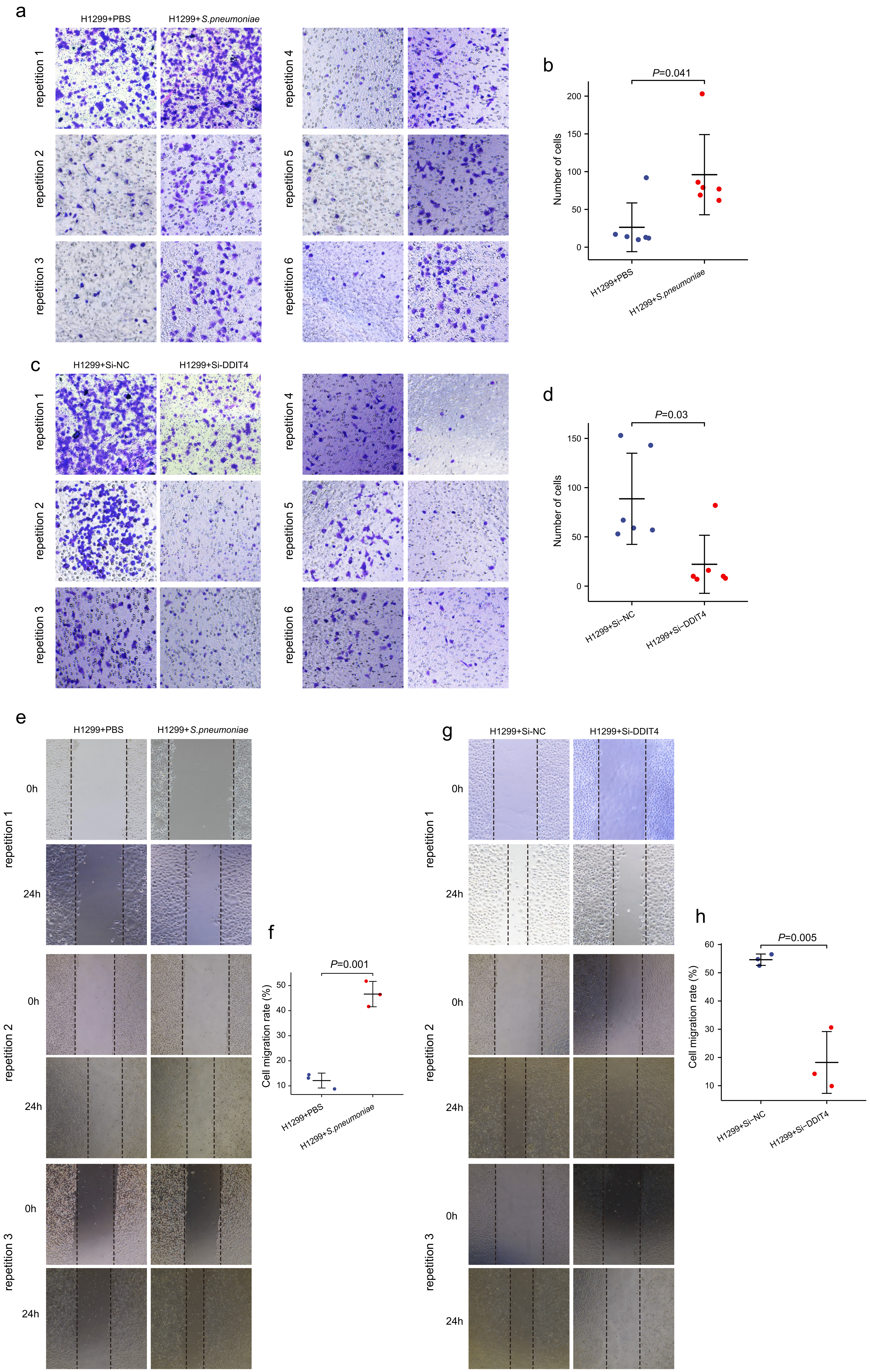
a
H1299+PBS
H1299+S.pneumoniae
repetition 1
repetition 2
repetition 3
repetition 4
repetition 5
repetition 6
b
P=0.041
Number of cells
200
150
100
50
0
H1299+PBS
H1299+S.pneumoniae
c
H1299+Si-NC
H1299+Si-DDIT4
repetition 1
repetition 2
repetition 3
repetition 4
repetition 5
repetition 6
d
P=0.03
Number of cells
150
100
50
0
H1299+Si−NC
H1299+Si−DDIT4
e
H1299+PBS
H1299+S.pneumoniae
repetition 1
repetition 2
repetition 3
0h
24h
f
P=0.001
Cell migration rate (%)
50
40
30
20
10
H1299+PBS
H1299+S.pneumoniae
g
H1299+Si-NC
H1299+Si-DDIT4
repetition 1
repetition 2
repetition 3
0h
24h
h
P=0.005
Cell migration rate (%)
60
50
40
30
20
10
H1299+Si−NC
H1299+Si−DDIT4

Figure S8. Effects of *S.pneumoniae* and DDIT4 expression on the migration and invasive abilities of H1299 cells. **(a)** Transwell migration assays of H1299 cells co-cultured with PBS and live *S. pneumoniae*. **(b)** Mann-Whitney U test for the results of six experimental replications. **(c)** Transwell migration assays of H1299 cells with silencing of DDIT4 and blank control. **(d)** Mann-Whitney U test for the results of six experimental replications. **(e)** Cell scratch assays of H1299 cells co-cultured with PBS and live *S. pneumoniae* for 0 and 24 hours, respectively. **(f)** The independent samples t test for the results of three experimental replications. **(g)** Cell scratch assays of H1299 cells with silencing of DDIT4 and blank control at 0 and 24 hours, respectively. **(h)** The independent samples t test for the results of three experimental replications.

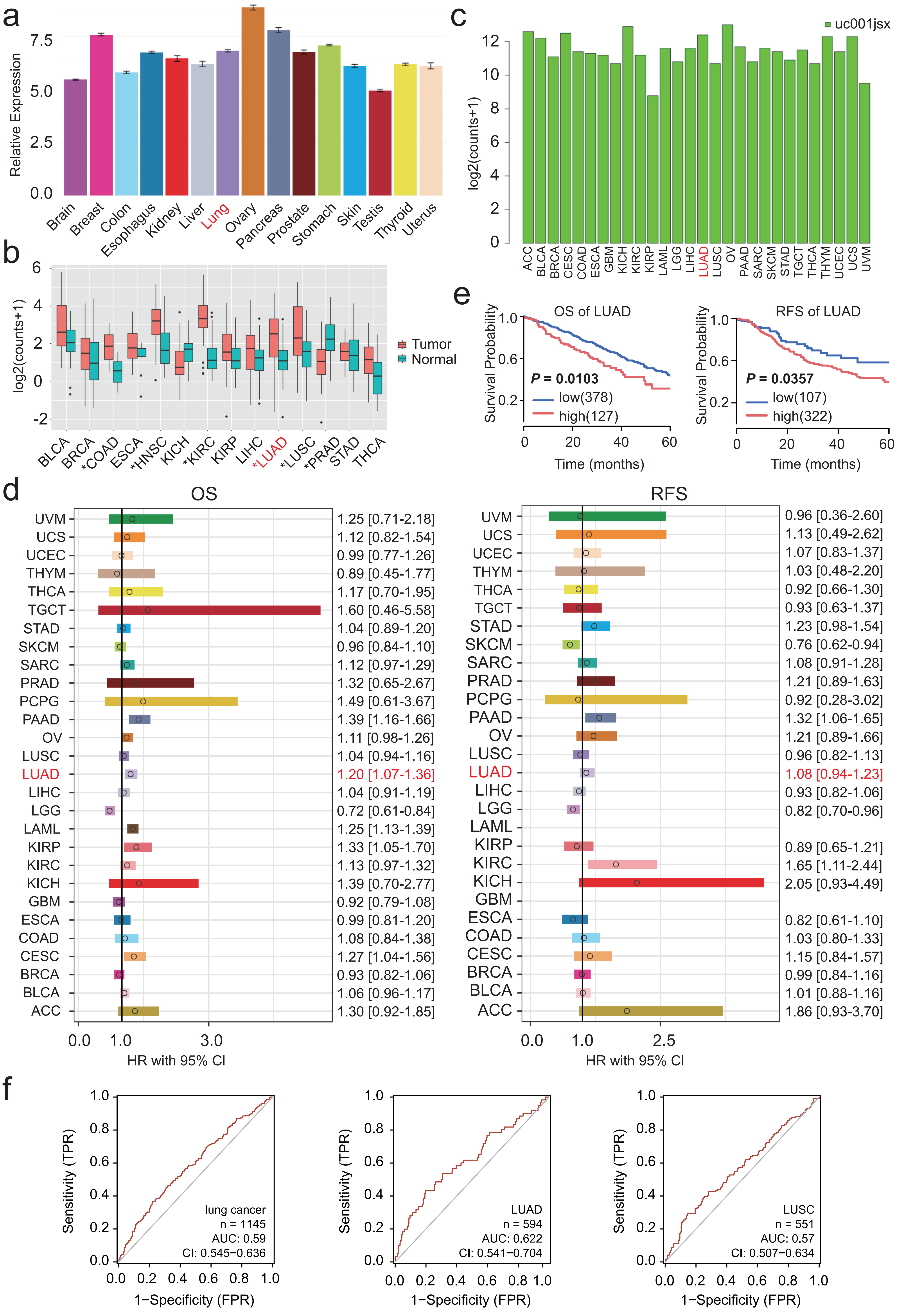
a
Relative Expression
0.0
2.5
5.0
7.5
Brain
Breast
Colon
Esophagus
Kidney
Liver
Lung
Ovary
Pancreas
Prostate
Stomach
Skin
Testis
Thyroid
Uterus
b
log2(counts+1)
6
4
2
0
-2
Tumor
Normal
BLCA
BRCA
*COAD
ESCA
*HNSC
KICH
*KIRC
KIRP
LIHC
*LUAD
*LUSC
*PRAD
STAD
THCA
c
uc001jsx
log2(counts+1)
0
2
4
6
8
10
12
ACC
BLCA
BRCA
CESC
COAD
ESCA
GBM
KICH
KIRC
KIRP
LAML
LGG
LIHC
LUAD
LUSC
OV
PAAD
SARC
SKCM
STAD
TGCT
THCA
THYM
UCEC
UCS
UVM
e
OS of LUAD
Survival Probability
P = 0.0103
low(378)
high(127)
Time (months)
RFS of LUAD
P = 0.0357
low(107)
high(322)
Time (months)
d
OS
UVM 1.25 [0.71-2.18]
UCS 1.12 [0.82-1.54]
UCEC 0.99 [0.77-1.26]
THYM 0.89 [0.45-1.77]
THCA 1.17 [0.70-1.95]
TGCT 1.60 [0.46-5.58]
STAD 1.04 [0.89-1.20]
SKCM 0.96 [0.84-1.10]
SARC 1.12 [0.97-1.29]
PRAD 1.32 [0.65-2.67]
PCPG 1.49 [0.61-3.67]
PAAD 1.39 [1.16-1.66]
OV 1.11 [0.98-1.26]
LUSC 1.04 [0.94-1.16]
LUAD 1.20 [1.07-1.36]
LIHC 1.04 [0.91-1.19]
LGG 0.72 [0.61-0.84]
LAML 1.25 [1.13-1.39]
KIRP 1.33 [1.05-1.70]
KIRC 1.13 [0.97-1.32]
KICH 1.39 [0.70-2.77]
GBM 0.92 [0.79-1.08]
ESCA 0.99 [0.81-1.20]
COAD 1.08 [0.84-1.38]
CESC 1.27 [1.04-1.56]
BRCA 0.93 [0.82-1.06]
BLCA 1.06 [0.96-1.17]
ACC 1.30 [0.92-1.85]
0.0 1.0 3.0
HR with 95% CI
RFS
UVM 0.96 [0.36-2.60]
UCS 1.13 [0.49-2.62]
UCEC 1.07 [0.83-1.37]
THYM 1.03 [0.48-2.20]
THCA 0.92 [0.66-1.30]
TGCT 0.93 [0.63-1.37]
STAD 1.23 [0.98-1.54]
SKCM 0.76 [0.62-0.94]
SARC 1.08 [0.91-1.28]
PRAD 1.21 [0.89-1.63]
PCPG 0.92 [0.28-3.02]
PAAD 1.32 [1.06-1.65]
OV 1.21 [0.89-1.66]
LUSC 0.96 [0.82-1.13]
LUAD 1.08 [0.94-1.23]
LIHC 0.93 [0.82-1.06]
LGG 0.82 [0.70-0.96]
LAML
KIRP 0.89 [0.65-1.21]
KIRC 1.65 [1.11-2.44]
KICH 2.05 [0.93-4.49]
GBM
ESCA 0.82 [0.61-1.10]
COAD 1.03 [0.80-1.33]
CESC 1.15 [0.84-1.57]
BRCA 0.99 [0.84-1.16]
BLCA 1.01 [0.88-1.16]
ACC 1.86 [0.93-3.70]
0.0 1.0 2.5
HR with 95% CI
f
Sensitivity (TPR)
1−Specificity (FPR)
lung cancer
n = 1145
AUC: 0.59
CI: 0.545−0.636
LUAD
n = 594
AUC: 0.622
CI: 0.541−0.704
LUSC
n = 551
AUC: 0.57
CI: 0.507−0.634

Figure S9. Tissue-specific expression of DDIT4 and its effect on prognosis. **(a)** Gene expressions of DDTI4 across normal tissues of GTEx data. **(b)** Different expressions of DDIT4 across 14 cancer types with more than ten samples between matched tumors and normals (TCGA). *significantly differentially expressed cancer types (|Fold change|>1 and FDR<0.05). **(c)** Gene isoform expressions of DDIT4 across 28 cancer types (TCGA). **(d)** Forest plot of Cox proportional HR and 95% CI of OS and RFS using 28 cancer types (TCGA). **(e)** Kaplan-Meier plots with log-rank tests of OS and RFS in LUAD (TCGA). **(f)** ROC curve analysis of DDIT4 as a prognostic indicator for lung cancer, LUAD and LUSC.

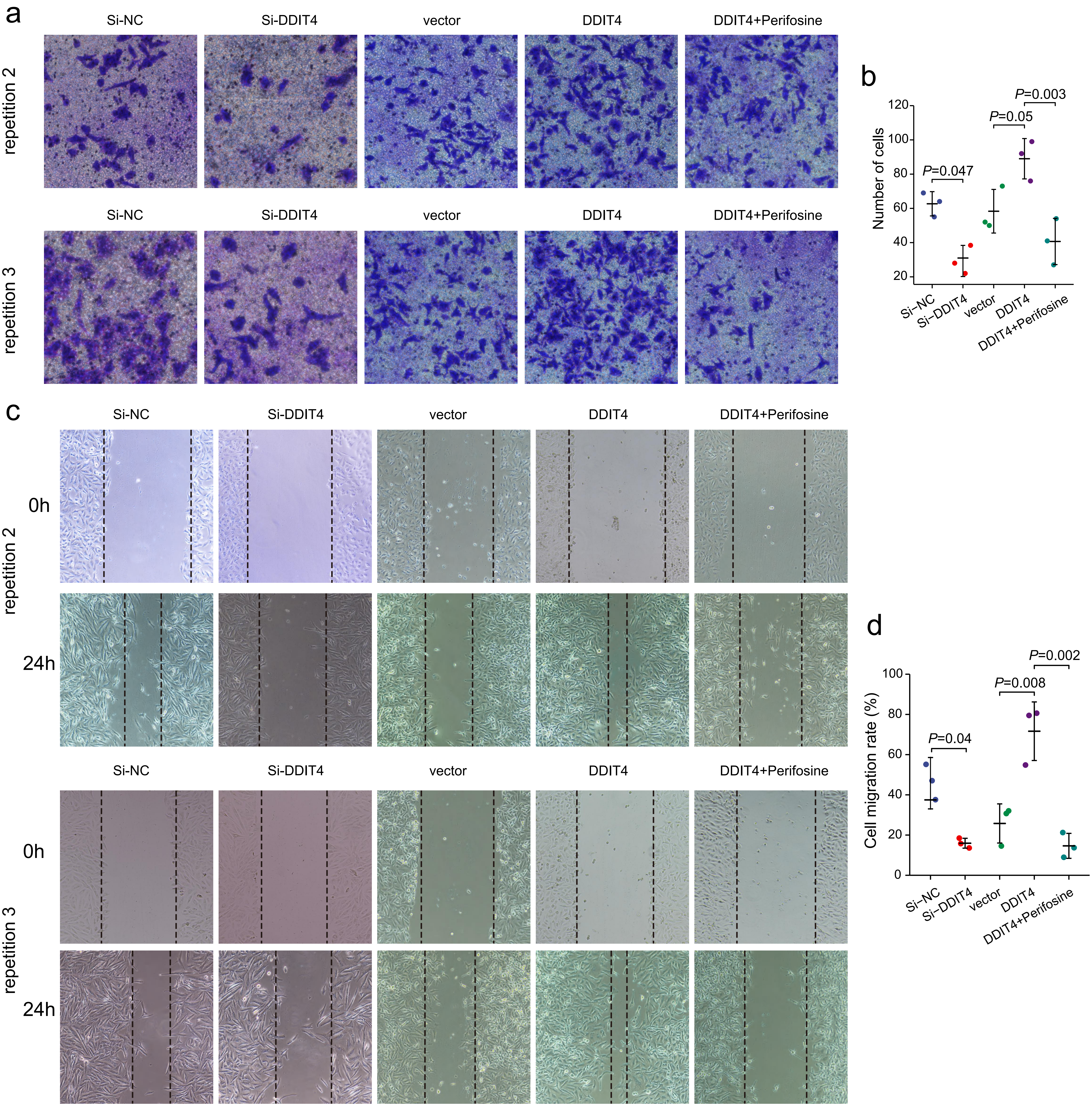
a
Si-NC
Si-DDIT4
vector
DDIT4
DDIT4+Perifosine
repetition 2
repetition 3
b
Number of cells
P=0.047
P=0.05
P=0.003
Si−NC
Si−DDIT4
vector
DDIT4
DDIT4+Perifosine
c
0h
24h
d
Cell migration rate (%)
P=0.04
P=0.008
P=0.002

Figure S10. High expression of DDIT4 enhances the migratory and invasive abilities of A549 cells. **(a)** Transwell migration assays of A549 cells with silencing of DDIT4, overexpression of DDIT4, overexpression of DDIT4 followed by addition of AKT inhibitor perifosine, and blank control. **(b)** One-way ANOVA and Tukey HSD Post hoc tests for the results of three experimental replications. **(c)** Cell scratch assays of A549 cells with silencing of DDIT4, overexpression of DDIT4, overexpression of DDIT4 followed by addition of AKT inhibitor perifosine, and blank control at 0 and 24 hours, respectively. **(d)** One-way ANOVA and Tukey HSD Post hoc tests for the results of three experimental replications.

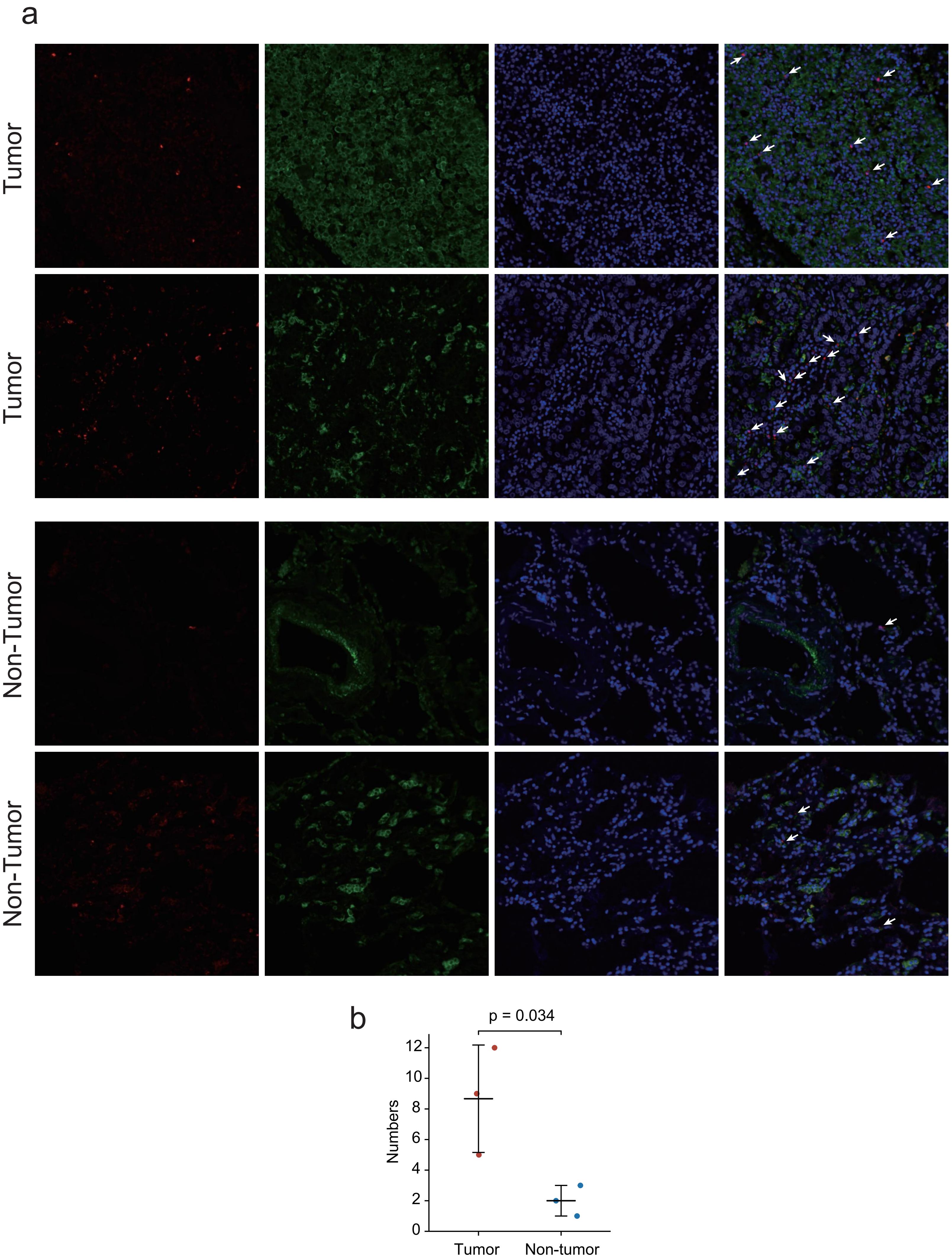
a
Tumor
Tumor
Non-Tumor
Non-Tumor
b
p = 0.034
Numbers
0
2
4
6
8
10
12
Tumor
Non-tumor

Figure S11. **(a)** Expression and enrichment of DDIT4 and *S. pneumoniae* in the other two lung cancer tissues and two paracancerous tissues. The white arrows refer to visible S. pneumoniae. **(b)** T-test results of the two groups of samples.
